# Engineered bacterial outer membrane vesicles: a versatile bacteria-based weapon against gastrointestinal tumors

**DOI:** 10.7150/thno.85917

**Published:** 2024-01-01

**Authors:** Keshuang Zheng, Yongpu Feng, Lei Li, Fanyang Kong, Jie Gao, Xiangyu Kong

**Affiliations:** 1 National Key Laboratory of Immunology and Inflammation, Naval Medical University, Shanghai, 200433, China.; 2 Institute of Neuroscience, Key Laboratory of Molecular Neurobiology of the Ministry of Education and the Collaborative Innovation Center for Brain Science, Naval Medical University, Shanghai, 200433, China.; 3 Department of Gastroenterology, Changhai Hospital, Naval Medical University, Shanghai, China.; 4 Digestive Endoscopy Center, Shanghai Tenth People's Hospital, Shanghai, China.; 5 Changhai Clinical Research Unit, Changhai Hospital, Naval Medical University, Shanghai, China.

**Keywords:** outer membrane vesicles, gastrointestinal tumors, genetic engineering, cargo delivery, tumor vaccine

## Abstract

Outer membrane vesicles (OMVs) are nanoscale lipid bilayer structures released by gram-negative bacteria. They share membrane composition and properties with their originating cells, making them adept at traversing cellular barriers. These OMVs have demonstrated exceptional membrane stability, immunogenicity, safety, penetration, and tumor-targeting properties, which have been leveraged in developing vaccines and drug delivery systems. Recent research efforts have focused on engineering OMVs to increase production yield, reduce cytotoxicity, and improve the safety and efficacy of treatment. Notably, gastrointestinal (GI) tumors have proven resistant to several traditional oncological treatment strategies, including chemotherapy, radiotherapy, and targeted therapy. Although immune checkpoint inhibitors have demonstrated efficacy in some patients, their usage as monotherapy remains limited by tumor heterogeneity and individual variability. The immunogenic and modifiable nature of OMVs makes them an ideal design platform for the individualized treatment of GI tumors. OMV-based therapy enables combination therapy and optimization of anti-tumor effects. This review comprehensively summarizes recent advances in OMV engineering for GI tumor therapy and discusses the challenges in the clinical translation of emerging OMV-based anti-tumor therapies.

## Introduction

Gastrointestinal (GI) tumors, comprising pancreatic ductal adenocarcinoma (PDAC), hepatocellular carcinomas (HCC), esophageal cancer, gastric cancer, and colorectal cancer (CRC), and so on, constitute a substantial proportion of both total cancer cases and cancer-related deaths, accounting for 26% and 35% respectively, on a global scale 1. In recent times, immunotherapy has manifested marked advantages and efficacy in treating malignancies, in addition to conventional therapies like surgery, radiation, chemotherapy, and targeted therapy. Presently, the most commonly employed immunotherapeutic agents primarily comprise checkpoint inhibitor monoclonal antibodies directed against programmed cell death 1 (PD-1) or its ligand, PD-L1, and chimeric antigen receptor T-cell (CAR-T) therapies, with other agents currently undergoing clinical trials 2. Despite the significant advancements in immunotherapy, its effectiveness and safety remain subject to reservations, thereby limiting its clinical utility in treating certain GI tumors 3, such as PDAC. Statistically, more than 99% of patients with PDAC are unresponsive to monotherapy with any of the currently approved immunotherapeutic agents by the U.S. Food and Drug Administration (FDA)-approved immunotherapeutic agents, except for a small fraction of patients with high microsatellite instability. The overall survival rate at 5 years in patients with PDAC in the U.S. is only around 9% 4. While immune checkpoint inhibitor-based therapy has displayed encouraging results in treating CRC, only a minute fraction of CRC patients with defective mismatch repair and high microsatellite instability have demonstrated any tangible benefits from it 5. Immunotherapy has showcased suboptimal outcomes in treating advanced CRC and HCC, with individual variability also affecting the therapy's efficacy. Consequently, there exists a pressing need for novel immunotherapeutic approaches such as improved drug delivery methods, as well as the identification and presentation of neoantigens, to treat GI tumors effectively.

**Figure 1:**
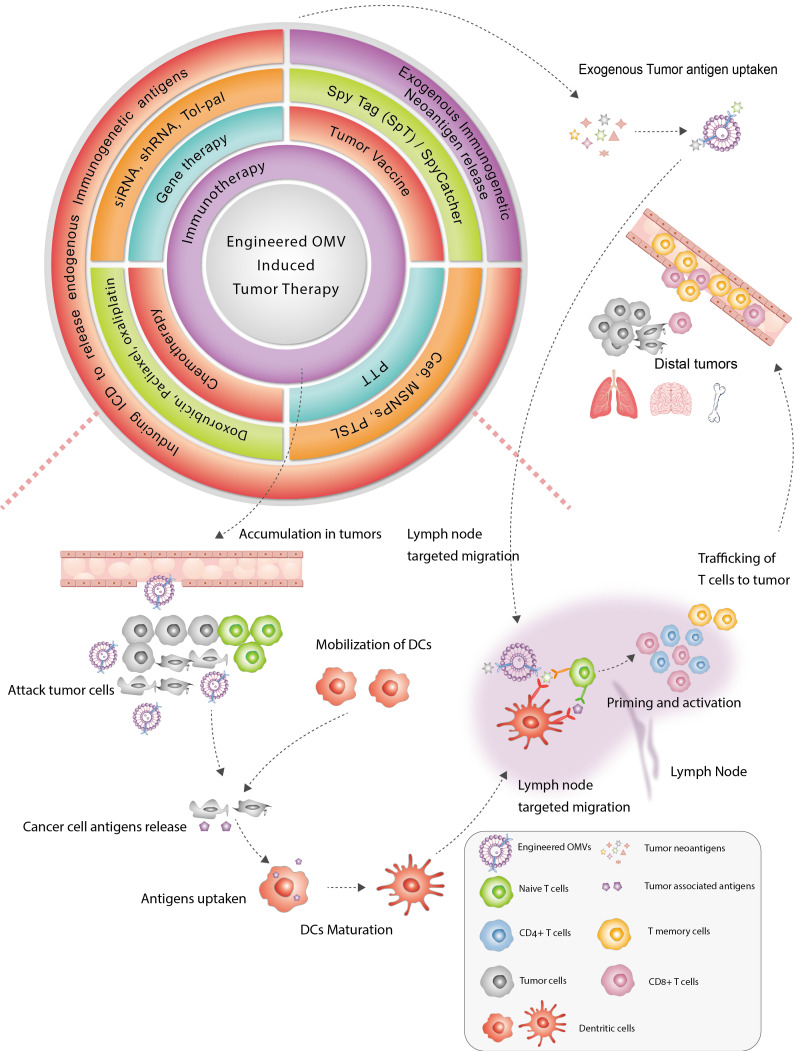
Summary of engineered OMV-based anti-tumor treatment. Immunotherapy is important in the field of OMV-based cancer therapy. In addition to immunotherapy, OMVs have been applied in combination with chemotherapy, gene therapy, and photothermal therapy to amplify anti-tumor efficacy. Engineered OMVs improved tumor immunogenicity by 1) delivering exogenous immunogenic antigens that mainly target lymph nodes to promote DC maturation and 2) inducing immunogenic cell death, to release endogenous immunogenic agents to promote DC maturation. Both strategies could promote T-cell priming and clonal expansion of T cells, leading to the suppression of both orthotopic and distal tumors.

Recent research has shed light on the potential of bacteria in the realm of cancer therapy. Bacteria exhibit inherent motility in that they colonize tumors preferentially and modulate the tumor immuno-microenvironment, thereby impacting the efficacy of immunotherapy 1. The utilization of bacteria themselves for the treatment of solid tumors dates back to the mid-19th century. However, the toxicity of bacteria presents a challenge in managing unnecessary cytotoxicity and mortality during treatment. Extracellular vesicles are a heterogeneous group of cell-derived lipid-bound structures, which involved in multiple physiological and pathological processes. Bacterial membrane vesicles, a form of extracellular vesicles secreted by bacteria, play a vital role in various biological functions of bacteria, including bacterial pathogenesis, interspecies communication and competition, oxidative stress, biofilm formation, and, significantly, regulation of the tumor microenvironment (TME) of GI tumors 6. Generally, extracellular vesicles from gram-negative bacteria are defined as outer membrane vesicles (OMVs), while those from gram-positive bacteria and eukaryotic vesicles are defined as membrane vesicles and exosomes, respectively 7. The OMVs could be observed at any stage of gram-negative bacteria 8. The GI tract, which is a vast reservoir of gram-negative bacteria, is a significant source of various OMVs in humans. Previous studies have demonstrated that OMVs from GI bacteria could enter intestinal epithelium sufficiently through various mechanisms to exert pathogenic or non-pathogenic effects 9. Given the impact on local immunity of the intestine and systematic immunity efficacy, OMVs are suitable for oral administration and have natural advantages in the treatment of GI tumors. Several studies have utilized OMVs to deliver drugs with some therapeutic efficacy in experimental animal models. OMVs possess several advantages over traditional drug delivery carriers, including greater drug-loading space and stability, higher biocompatibility, appropriate immunogenicity, and lower cytotoxicity. Despite the intrinsic immune adjuvant properties, the natural anti-tumor effects of them alone are limited. Recently, engineering modifications of OMVs have been developed to endow extracellular vesicles with new properties through genetic engineering or physicochemical methods, aimed at improving the yield, safety, and targeting capability of OMVs during drug delivery. Notably, the promising strategies combined with nanotechnology are able to evoke potent tumor-specific immune responses, inhibiting tumor growth and metastasis (Figure 1). In conclusion, engineering strategies provide great opportunities for improving the efficacy of tumor treatment.

As an emerging class of immune-oncology therapy, tumor vaccines attack tumor cells via specific cytotoxic T-cells lymphocytes (CTLs), which are activated by tumor antigens. Oral vaccines should elicit robust anti-tumor immune responses since the intestine acts as the largest immune organ, containing about 70% of the immune cells of the body. OMVs derived from commensal bacteria assemble natural epithelial penetration and good oral tolerability, as well as the underlying roles in modulating the gut microbiota, mucosal adaptive immune responses, and the physicochemical barrier in assisting anti-tumor therapy. Increasingly researchers have spotlighted the potential of using gut microbiota-derived OMVs as an alternative to artificial nano-materials, for safer and more effective oral GI tumor vaccine. For instance, some studies have demonstrated that oral administration of *Akkermansia muciniphila* (Akk) OMV enhances immune checkpoint blockade (ICB) therapy against GI tumors by maintaining intestinal homeostasis, reprogramming the TME, as well as promoting CTL-related immune response 10,11.

Over the past decade, some reviews have reported progress in the application of engineered OMVs in anti-tumor drug delivery and immunotherapy 12-15. However, to the best of our knowledge, few reviews have specifically focused on the potential application of engineered OMVs in the treatment of GI tumors. Based on the literature, our review covered common engineering strategies including genetic modification, drug-loading, surface modification, as well as biomimetic nanoparticles (NPs). Moreover, the advantages and disadvantages of engineered OMVs for the treatment of GI tumors have also been discussed. Finally, the challenges associated with these emerging OMVs platform-mediated anti-tumor therapies and their clinical translation are mentioned, which will help to better understand the current advance and future research directions in this field.

## 1. Overview of OMVs

### 1.1 Structure and biogenesis of OMVs

OMVs are non-replicative spherical nanovesicles ranging from 20-250nm, which consist of a typical phospholipid bilayer naturally derived by Gram-negative bacteria. The vesicles inherited components from bacterial membranes, presenting excellent intrinsic immunostimulatory properties. Besides some physiological processes including intracellular and extracellular communication, quorum sensing, and horizontal gene transfer, OMVs are ascribed to many biological functions such as ligand recognition, and biological targeting. Further, OMVs have been shown to be highly stable even upon elevated temperatures and several harsh environmental conditions 16. The outer membrane of OMVs is composed of phospholipids, lipopolysaccharides (LPS), and outer membrane proteins (OMPs). The stability of the membrane structure is co-maintained by the covalent crosslinking between OMPs and lipoproteins (Lpps), as well as the non-covalent crosslinking between outer membrane pore proteins and the peptidoglycan (PG) layer that located in the periplasmic space. The membrane proteins of OMVs include OMPs, soluble periplasmic proteins, and the Tolerance peptidoglycan-associated lipoprotein (Tol-Pal) complex, which consists of TolA, TolB, TolQ, TolR, and Pal. Tol-Pal complexes are partially anchored to the inner membrane of OMVs, cross the periplasmic space, and are connected to the PG layer and the outer membrane of OMVs by noncovalent bonds. The absence of any component of Tol-Pal and any factor that destroys the cross-linkage between PG and Lpp or OMP may change the stability of the OMVs membrane and thus trigger OMVs vesiculation 17. Although there are no definite conclusions regarding the biogenesis of OMVs, several biogenesis pathways have been reported. The basic principles for membrane curvature induction include (1) Selective local protein and cargo protein crowding in the periplasm or outer membrane. (2) Insertion of the Pseudomonas quinolone sequence (PQS) increases the surface area of the outer leaflet relative to the inner leaflet by a wedging effect 18. (3) Specific enrichment of different fatty acids and LPS in some areas. (4) Phospholipids accumulation in the outer membrane in the absence of the VacJ/Yrb ATP-binding cassette (ABC) transport system. All models depend on an initial decoupling of the outer membrane by breakage of the Lpp crosslink loss, even this decoupling alone is sufficient to induce membrane vesiculation.

### 1.2 Preparation of OMVs

High OMV quality and purity are required in bringing OMV into the clinical setting. To meet this requirement, several methods have been developed to isolate and purify OMVs. Density- or size-based isolation is the most widely used method for OMV preparation that includes ultracentrifugation, ultrafiltration, tangential flow filtration, *etc.* These methods are usually simple in procedures but get OMV in limited purity. Another method is affinity-based OMV isolation, which collects OMVs according to special ligands presenting on the surface, such as antibodies, aptamers, and resin. These techniques isolate OMV in high purity from the culture broth. Nevertheless, the process of affinity isolation is time-consuming and results in product loss.

To meet the requirement for OMV basic and clinical research, Liu* et al.* have summarized a set of preparation protocols for the vast majority of bacteria, which has been used for *E. coli Nissle 1917* and *Akk* OMV separation successfully 19. Firstly, the culture medium containing bacteria and their debris is usually removed by low-speed centrifugation and a 0.22μm sterile filter. Secondly, the small molecule proteins are eliminated by ultrafiltration (100 kDa). Further, the OMVs are purified by ultracentrifugation as well as iodixanol gradient centrifugation. Finally, OMVs should be characterized by transmission electron microscopy, nanoparticle tracking analysis, or western blotting, if necessary 20. These steps mentioned above achieve sufficient yield and purity in most cases. However, combining different techniques is imperative in OMVs isolation in complex media, such as biofluids 20. Different methods could hamper the repeatability and reproducibility of outcomes between researchers. To promote in-depth studies of OMVs and their clinical translation, we dire need standardized guidelines that take cost, efficiency, and quality into consideration at the same time 20.

### 1.3 Internalization of OMVs

In GI tumors, OMVs have been reported to interact directly with epithelial cells at mucosal surfaces, immune cells as well and other host cells including endothelial cells, platelets, osteoblasts, and synovial cells. Several mechanisms have been put forward for OMV uptake into host cells, which can be roughly classified into 2 types. The first one is receptor-mediated OMV internalization. OMVs can bind to certain receptors, such as clathrin, caveolin, or lipid raft, and then activate receptor-induced intracellular signaling in recipient cells. OMVs of* Porphyromonas gingivalis* and *Helicobacter pylori* (*H. pylori*) utilized clathrin-mediated endocytosis to gain entry into GI epithelial cells 21. Later, Kaparakis* et al.* declared that *H. pylori* OMVs enter GI tumor cells via both clathrin and caveolin-mediated endocytosis, with a preference for dynamin-dependent and caveolin-mediated endocytosis 22. Subsequently, O'Donoghue* et al*. reported that *H. influenzae*,* M. catarrhalis*, *V cholerae*, as well as *Enterotoxigenic Escherichia Coli* (*ETEC*) OMVs, invade GI epithelial cells mainly via caveolin-mediated endocytosis 14,22. Another mechanism of OMV internalization is direct fusion to host cell membranes. OMV fusion to host cell lipid rafts induces actin remolding to allow OMV soluble cargo to diffuse directly into the host cytoplasm. Lipid rafts-mediated entry has been observed in *V cholerae*, *P. Aeruginosa*,* C. jejuni, A*.* baumannii*, and *P. gingivalis etc*. 21*.* Membrane fusion is used by *L. pneumophila, P. Aeruginosa*, and *A. actinomycetemcomitans* OMVs. As a special type of receptor-independent OMV Internalization, macropinocytosis is described as the inward folding by some of the cell surface ruffles to fuse with the basal membrane, which has been observed in OMVs from *Shigella flexneri* internalized by the human epithelial cells and fibroblasts 23. This is also the mechanism proposed for *P. aeruginosa* OMVs' interaction with the airway epithelial cells 24. As described above, the pathway employed is organism-, or even strain-dependent. Moreover, the size of the OMV population is relevant when studying endocytic routes. Clathrin-mediated endocytosis generally allows the internalization of larger cargo than clathrin-independent routes, while the macropinocytosis allows internalization of endocytic vesicles up to 1 um in diameter 25.

### 1.4 Biological characteristics of OMVs

Based on previous studies, OMVs showed a more rigid drug package as well as larger drug loading space when compared to normal liposomes 26. Further, OMVs exhibit high environmental stability at higher ambient temperatures and a wide range of pH values. Consequently, OMVs safeguard their payload from enzymatic degradation during long-distance drug delivery *in vivo* without obvious leakage in systemic circulation. Alves* et al.* successfully packaged the enzyme phosphotriesterase (PTE) into the lumen of *E. coli*-derived OMVs and observed enhanced stability of OMVs-encapsulated PTE relative to free PTE after numerous freeze-thaw cycles 26. Their subsequent study showed that *E. coli*-derived OMV-encapsulated PTE protects enzyme activity in harsh environments, such as heating and freeze-drying 27. In addition, OMVs derived from *Salmonellae* and *Shigella* contain adhesins that enable themselves to be recognized and endocytosed by the GI tract cells without any targeting ligands assembled 25. These studies efficiently underscore the remarkable features of OMVs as a platform for anti-tumor treatment.

**Figure 2:**
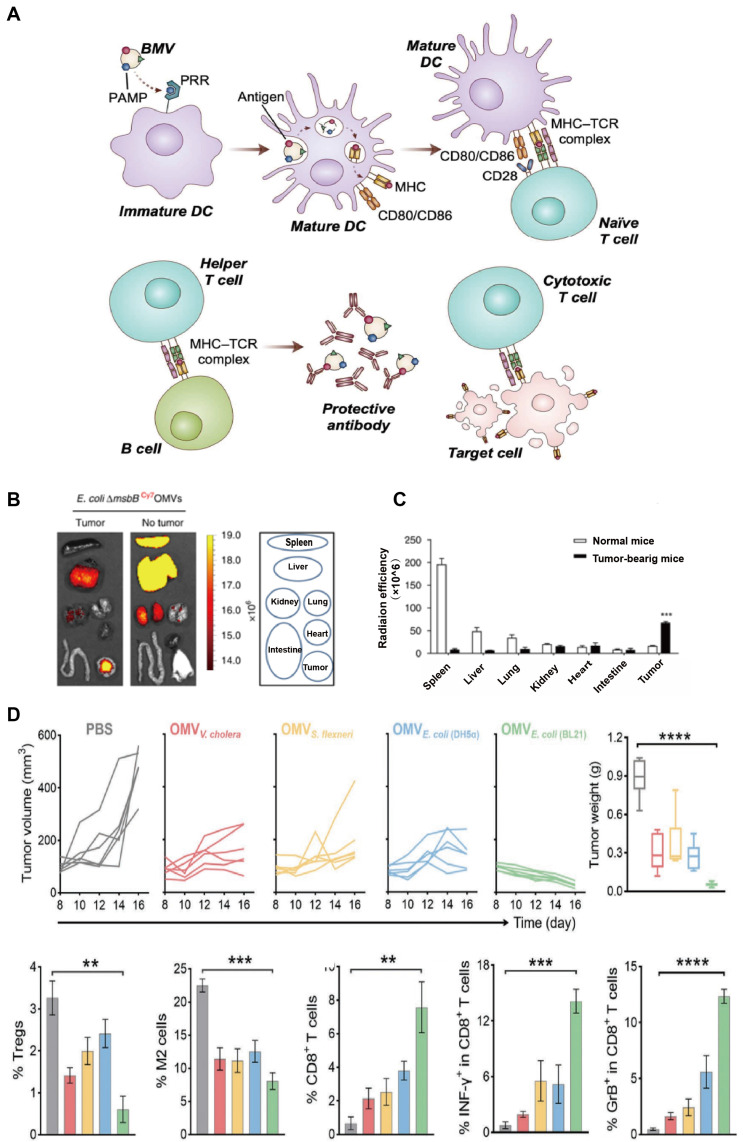
The safety, immunomodulatory and anti-tumor effects of OMVs. A) OMVs containing various PAMPs that promote DC maturation following interaction with PRRs. Mature DCs elicit the proliferation and activation of antigen-specific CD4+ T and CD8+ T cells in lymph nodes. Adapted with permission from 34, copyright 2022 Advanced Drug Delivery Reviews. B) *In vivo* fluorescence images of CT26 tumor-bearing mice and tumor-free mice post *i.v,* injection of OMV@Cy7. The circles indicate different organs.* Ex vivo* imaging showing the distribution of OMV@Cy7 in the major organs and tumor sites at 24 hours post injection. C) Quantitative analysis of the Cy7 fluorescence intensity in the spleen, liver, heart, kidney, lung, intestine, and tumor. D) The CT26 mice were intravenous injected with OMVs three times every other day. Individual tumor growth kinetics after treatment with different types of OMVs and their tumor weights, as well as percentage of Tregs in tumor tissues were recorded. Adapted with permission from 35, copyright 2020 Advanced Materials.

Moreover, immunogenecity is the most prominent property of OMV in its application in tumor treatment. OMV can be identified and phagocytosed by antigens to antigen-presenting cells (APCs) more easily than other nanostructures (*e.g.,* liposomes). The LPS presenting on the OMVs surface can stimulate different pattern recognition receptors (PRRs), producing proinflammatory cytokines which are crucial for dendritic cell (DC) recruitment and maturation. Additionally, the spherical nanoscale structure of OMV stimulates APCs to present tumor antigen, thus provoking both antigen-specific B-cell and CTL (Figure 2A). Some studies have examined the interaction between OMVs and DCs. OMVs from *Streptococcus* were observed rapidly taken up by the DC with increased TNF-α releasing 28. In another research, the intraperitoneal injection of *Salmonellae*-derived OMVs into mice elicited an increase in the expression of CTLs along with a high level of TNF-α in the spleen 29. All of the above evidence indicates the potential of OMVs in enhancing antigen presentation and immune responses in GI tumors (Table 1).

## 2. Engineered OMV for GI tumor therapy

Compared with live or attenuated bacteria, native OMVs are considered safer since they cannot replicate autonomously *in vivo* 36. Furthermore, nano-sized bacterial native OMVs can penetrate various cellular barriers and evade clearance by the immune system more easily than the bacteria. Nevertheless, emerging evidence reflected shortcomings of native OMV in tumor therapy recently. Kim* et al*. labeled OMVs with Cyanine7 (Cy7) and detected different levels of OMVs aggregation in the GI tract, liver, and heart, even though the highest signal was detected in tumor sites 12 hours after intravenous injection (Figure 2B-C). Native OMVs may exhibit off-target effects, thereby leading to unpredictable effects on sites other than tumor tissues. Further, the native OMV-induced anti-tumor effect based on IFN-α was generally limited to tumor enlargement inhibition rather than tumor regression 35 (Figure 2D). To enhance the efficacy and reliability of OMVs in the treatment of GI tumors, there is a need to optimize their targeting ability and immunogenicity while also enhancing their safety and controllability through engineering approaches.

It has been found that genetic modification and physicochemical methods, also known as the engineering of OMVs, hold promise for overcoming these problems. Commonly used methods for modification include (i) Genetic engineering of source bacteria to obtain 'customized' OMVs. (ii) Loading of therapeutic drugs through electroporation, extrusion, ultrasonication, or coincubation. (iii) Combination of OMVs with nanocarriers to form biomimetic NPs. (iv) Surface modification of OMVs using physical or chemical methods to reduce adverse effects. Modification of OMVs often requires multiple approaches due to tumor heterogeneity and organism complexity. The three main engineering methods (i-iii) are categorized and discussed below, while surface modification of OMVs is introduced in each case in this article. Additionally, the progress of several primary cancer therapies using engineered OMVs such as tumor vaccines and photothermal therapy (PTT) will be discussed.

### 2.1 Genetic Modification of OMVs

Despite the demonstrated anti-tumor effects of OMVs in certain studies, natural OMVs typically contain an excessive amount of immunogenic substances, potentially leading to severe systemic inflammatory response syndrome upon intravenous administration 37. Moreover, the insufficient yield of OMVs poses a significant challenge to their clinical development and application. As such, there is a pressing need to explore and refine techniques for processing and modifying OMVs to address these issues.

#### 2.1.1 Optimization of the targeting ability of OMVs

**Figure 3:**
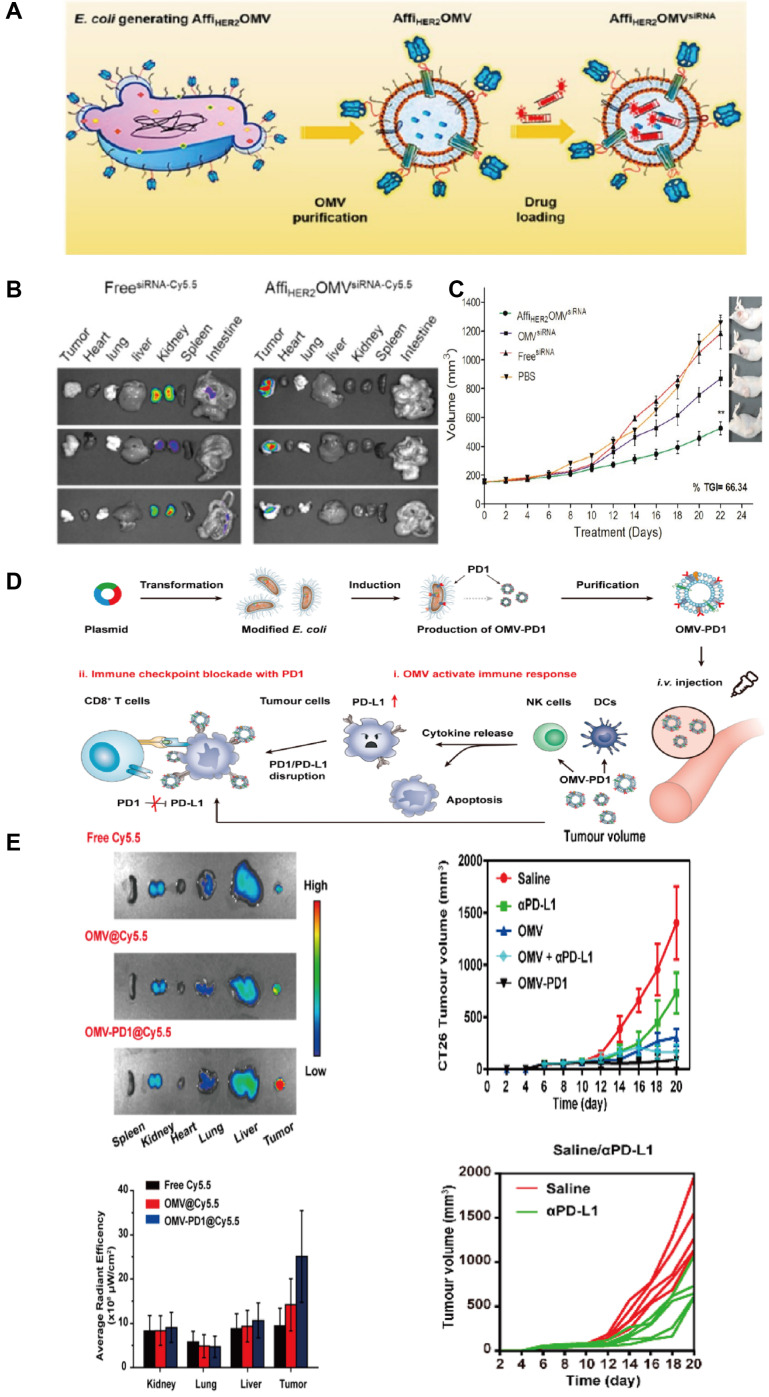
Anti-tumor effects of engineered OMVs with gene modifications. A) The OMV was purified after vesiculation from the parent bacteria that inserted with ClyA-Affibody and further loaded with siRNA using an electroporation method. B) For optical imaging, HCC-bearing mice were given a single injection of Cy5.5-labeled Affi*HER_2_*OMV^siRNA‑Cy5.5^, which shows the accumulation of OMVs in the whole body after systematic administration. C) The engineered OMVs exerted the strongest anti-tumor effects compared to all controls. Adapted with permission from 43, copyright 2013 ACS Nano. D) OMV-PD1 were obtained by engineering* E. coli* to stably express the PD1 ectodomain fused with the ClyA. OMV-PD1 accumulation at the tumor site increases the infiltration of immune cells. At the same time, the PD1 ectodomain on the OMV-PD1 and protects CD8+ T cells, which can then attack tumor cells. E) Tumor tissues and major vital organs (lung, liver, kidney) were analyzed separately. The images show tumor-specific retention and accumulation of engineered OMV delivery system. Engineered OMVs exerted the strongest anti-tumor effects compared to all controls. Adapted with permission from 44, copyright 2020 ACS Nano.

**Figure 4:**
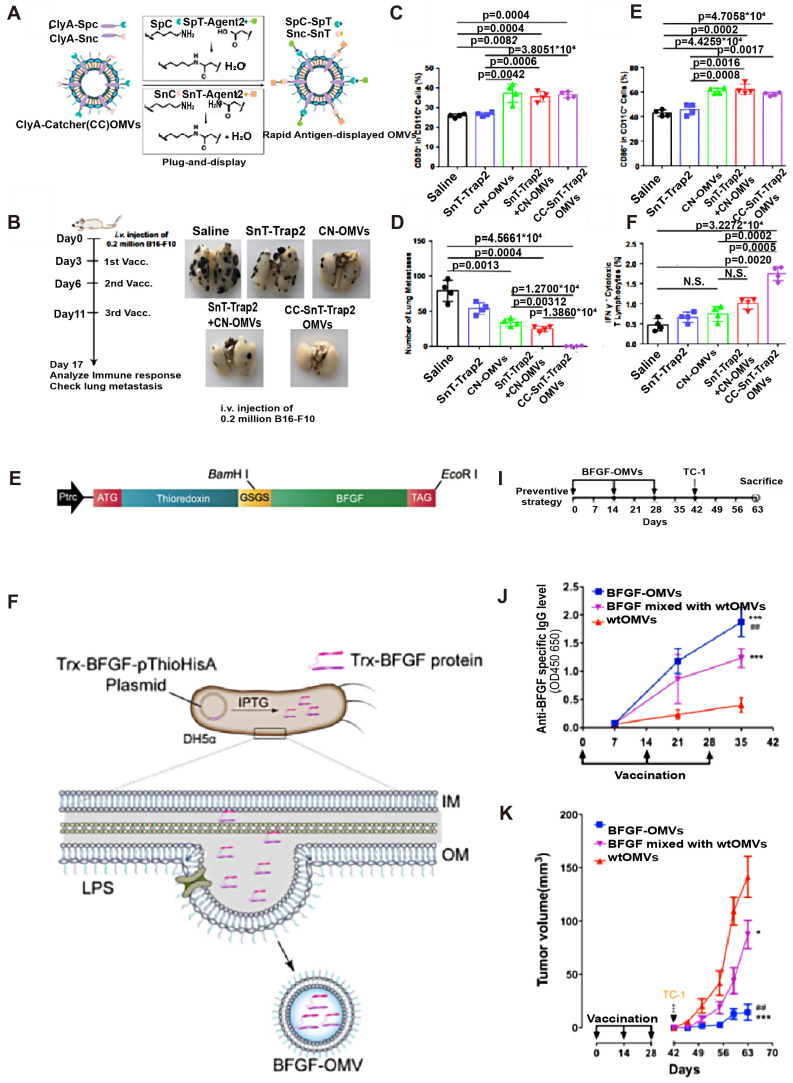
Anti-tumor effects of engineered OMVs with optimized immunogenicity. A) A versatile OMV-based vaccine platform can rapidly and simultaneously display multiple tumor antigens and consequently elicit synergistic anti-tumor immune responses for personalized tumor vaccines. B, D) Engineered OMV vaccination inhibits tumor metastasis. The percentage of CD80+(C) or CD86+ (E) cells in CD11c cells was determined by flow cytometry. F) The percentage of IFN-γ cells in the CD3+CD8+ T-cell subpopulation is shown. Adapted with permission from 28, copyright 2021 Nature Communication. G) Schematic diagram of gene recombination of Trx and the whole BFGF molecule. H) Schematic diagram of the principle of production of BFGF-OMVs. Trx guided BFGF to the periplasmic space. The fusion protein was coated during the process of OMV formation in bacteria. I) Schematic diagram of the procedures for the tumor preventive experiment with BFGF-OMVs J) Detection of the level of specific anti-BFGF autoantibodies in mouse serum using ELISA. K) Continuous measurements of tumor volume in mice. BFGF-OMVs significantly reduced tumor volume. Adapted with permission from 36. Copyright 2020, Acta Biomaterial.

Most of the research on tumor-targeting optimization adopts genetic modification. Human epidermal growth factor receptor 2(HER2) is overexpressed in some GI tumors (*e.g.* Gastric cancer) and plays an important role in tumor cell growth, survival, and differentiation, thus becoming a popular target for cancer diagnosis and treatment 38. Gujrati *et al*. fused HER2 ligands with Cytolysin A (ClyA), commonly expressed on *E. coli* OMVs, by genetic modification (Figure 3C). It was observed that engineered *E. coli* OMVs-specific accumulated in HER2-overexpressing tumor tissues after tail vein injection in an HCC mouse model 39 (Figure 3A). This was the initial* in vivo* study to use engineered OMVs for the purpose of optimizing the targeting ability of OMVs. In addition to molecular targeting, Li *et al*. combined engineered OMVs with ICB in CRC therapy and obtained OMV-PD1 40 (Figure 3E-F) by fusing the PD-1 coding region with the ClyA coding region of *E. coli*. As expected, a large number of immune cell infiltrates was observed in tumor tissues because of the OMV-PD1 induced PD-L1 blocked on the surface of tumor cells, which suggests that genetically engineered OMV-PD1 has reversed the immunosuppressive TME in CRC besides specific target. Another key target in ICB is the CD47 which is abnormally expressed on tumor cells that bind to signal-regulatory protein alpha (SIRPα) of macrophages, and responsible for evading phagocytosis. Feng *et al*. modified OMV with CD47 antibodies to block the CD47-SIRPα binding efficiently. Additionally, the OMV-CD47nb were coated with the PEG/Se layer, which enabled the OMV-CD47nb with X-ray-controlled tumor targeting 41. In another study that focused on targeted transportation of tumor APCs, Cheng *et al*. constructed a 'tumor antigen plug-and-display procedure' aiming to modify multiple tumor target antigens on the surface of OMVs (Figure 4A-F). On a SpyTag (SpT)/SpyCatcher (SpC) capture system, the SpT protein tag is used to label various tumor antigens, whereas SpC is an antigen catcher that binds to the OMVs surface protein ClyA. SpT with bound tumor antigens can bind to SpC via peptide bonds, thereby concentrating tumor antigens bound by SpT on the surface of engineered OMVs (Figure 4A). Subsequently, engineered OMVs carrying tumor antigens activate DCs in the TME and are redirected into peritumoral lymph nodes owing to their superior nanoscale size and immunogenicity. Ultimately, these OMVs induce a systemic, memory-based anti-tumor immune response *in vivo* 42. The anti-tumor efficiency of the OMV-based antigen capture system has been confirmed in mouse models, which is presented in Figure 4B-F. Collectively, genetic engineering enhances the tumor-targeting ability of OMVs and allows their integration with immunotherapeutic strategies, such as ICB therapy and tumor vaccines, to elicit a broader anti-tumor-immune response. Engineered OMVs, which act as tumor antigen-presentation systems, could avoid the loss of tumor antigens and exert potent tumor-killing effects.

#### 2.1.2 Optimization of the immunogenicity of OMVs

Native OMVs caused mild to moderate immune responses, most of which were not directed against the heterologous expressed antigens necessarily 45. To improve the tumor-targeting immune response, Zhang* et al.* engineered the factor H binding protein (fHbp) of *E. coli*, which increased both fHbp and antigen yields per OMV, thus improving the antibody responses against fHbp and target antigen. Likewise, Scatters* et al.* modified the surface of OMVs to express numerous ovalbumin (OVA) fragments by injecting plasmids containing OVA DNA sequences into *Salmonella typhi*. OVA fragments contain two antigenic epitopes capable of binding to MHC-I and MHC-II. After activation of DCs by engineered OMVs owing to their immunogenicity, the OVA fragments activated CD4+ T cells and CD8+ T cells, which in turn induced a persistent immune response 46.

The interaction of the OMV with the immune system can be considerably tuned. It has been proved that using monoclonal antibodies to block tumor angiogenesis is an effective Anti-tumor method. However, it is very expensive, time-consuming, and even easily induces drug resistance. Following the use of anti-vascular endothelial growth factor (VEGF) monoclonal antibodies such as bevacizumab, compensatory basic fibroblast growth factor (BFGF) expression promotes angiogenesis and resistance to targeted therapy. To address this issue, Huang *et al.* constructed a recombinant plasmid containing the *bfgf* gene transfected with *E. coli* (Figure 4G and H), thus resulting in spontaneous production of anti-BFGF immunoglobulins in CRC-bearing mice (Figure 4I-K) 47.

#### 2.1.3 Optimization of the yield of OMVs

OMVs have exhibited immense potential in the field of biomedical applications. However, the current production levels of OMVs are insufficient to meet the demand for their clinical translation. In response to this challenge, Lloubès* et al.* investigated to elucidate the role of bacterial membrane structural integrity in the production of OMVs. Through gene screening and analysis, the researchers discovered that mutations, either individually or combined, in certain genes from the Tol-Pal protein family led to an increase in OMV production 17. The Tol-Pal family, composed of five interacting proteins forming a cytoplasmic periplasmic space membrane protein complex, interacts with OMPs and Lpps in the bacterial outer membrane. Mutations in any of the five *Tol-Pal* genes lead to defects in the bacterial outer membrane, which result in elevated production of OMVs and the release of periplasmic proteins 17. Reimer* et al.* also reported a noteworthy increase in OMV production upon the deletion or mutation of the *tolA, tolR*, and *tolB* genes in *E. coli.*, which are keys in maintaining membrane stability 48. In addition, the deletion of *RmpM*, a member of the Lpps family and encoding an outer membrane lipoprotein covalently cross-linked with PG layer, loosens the outer membrane and promotes OMVs release 49. Waterbeemd *et al.* verified this principle through the knockout of the *RmpM* gene in *Neisseria meningitides*, which resulted in a significant increase in OMV production 37. According to McBroom* et al*., in addition to interventions on genes related to membrane integrity, affecting bacterial membrane stress pathways can also enhance OMV production. Specifically, they found a 100-fold increase in OMV production upon knocking out the *E. coli* ^σ^E stress pathway-related genes *degS* and *degP*, without any associated membrane integrity defects 50. Other identified genes such as the *nlpI* and the *ompR* were similarly linked to the cell envelope, affecting outer membrane protein expression and localization. The *waaG* and the *ponB* were related to LPS and PG biosynthesis and the deep and the *rseA* affected the σE envelope stress response pathway 51. It is conceivable that certain mutations that reliably force hyper-vesiculation might have unexpected benefits for different OMV applications. These findings underscore the role of gene modification in altering the production of OMVs via various pathways, thus enabling the clinical translation of engineered OMVs (Table 2).

A main shortcoming of genetic modulation is that one mutation may not work widely in all Gram-negative bacteria. Emerging evidence shows that exploring environmental triggers for OMV release could be a convenient way to enhance production. For example, Gerritzen* et al.* found that up-regulated dissolved oxygen tension in cysteine depleted cultivations resulted in *N. meningitidis* OMV double production. The increased yield may be caused by a reduced resistance to oxidative stress due to cysteine limitation 66. However, cysteine-depletion also leads to bacteria growth arrest and even accumulation of undesired components, like DNA, and ammonium that hinder further purification of OMV 67. Besides, Waterbeemd* et al.* found that sterile equipment provides better stability and higher yield OMVs because the filtration step removes most constituents that might inhibit growth 68. Recently, Gerritzen* et al.* combined sulfur source depletion with high dissolved oxygen and reached OMVs continuous production, which has been used clinically in the production of vaccines 69.

#### 2.1.4 Optimization of the biosafety of OMVs

One of the main considerations of OMVs application is safety. Traditional approaches principally use deoxycholate or sodium dodecyl sulfate to produce less toxic OMVs, such as OMV-based *N. meningitides* vaccines. While effective, the use of detergent partially decreased both Toll-like receptors (TLR) 4 and TLR2 activation induced by OMVs. Hence, novel strategies are required to manage the toxicity of OMVs while preserving their immune adjuvant effects. The mechanism of different OMVs initiates PRR signaling is highly heterogeneous. S Robbana-Barnat* et al.* have declared that both tri-acylated lipoprotein and bi-acylated lipoproteins can stimulate TLR2 responses, followed by TLR2 dimerization with TLR1 and TLR6 separately. Flagellin stimulates TLR5 responses, unmethylated bacterial CpG DNA stimulates TLR9 responses, and bacterial ribosomal RNA stimulates TLR13 responses. Moreover, one of the most important TLR for pathogen-associated molecular patterns (PAMPs) recognition is TLR4, which detects the lipid A of LPS 70.

A previous study reported that the use of *Neisseria meningitides* OMVs incorporating Hexa-acylated LPS as vaccines led to a severe, even lethal, inflammatory, while OMVs with the *lpxL1* gene deletion displayed significantly reduced toxicity. This could be ascribed to the production of Penta-acylated lipid A resulting from the altered biosynthetic pathway of LPS after the knockdown of *LpxL1* or *LpxL2* 71. Likewise, Xue* et al.* modified lipid A in *E. coli*, *Shigella*, and *Salmonellae* via the inactivation of genes encoding late acyltransferases (*e.g.*, de-acylase *LpxR*, *lpxM* or *PagL*), which causes lipid A structural rearrangements with decreased ligand affinity for the TLR4/MD-2 complex and subsequently reducing the inflammatory response 12. Alterations in the number of acyl chains and phosphate groups are commonly used methods, more studies on the regulation of OMVs virulence through the genetic modulation of bacteria are summarized in Table 3.

In fact, other explorations except for the genetic modification in reducing excessive OMV immunogenicity have also been undertaken. Qing* et al.* encapsulated OMVs with a highly biocompatible pH-sensitive shell of calcium phosphate, which overcame the severe systemic inflammation observed in naked OMVs after intravenous injection 35. Zheng* et al.* came up with bacterium-mimicking vectors by rearranging the PAMPs, which displayed optimized anti-tumor therapeutic and prophylactic effects 80. Further, LPS-neutralizing peptides could be used to reduce strong inflammatory responses induced by OMVs. Unfortunately, there is no universal peptide that can be applied to all OMVs. Selecting OMVs from commensal bacteria with lower immunogenicity or designing OMV-based oral formulations tend to be promising approaches, especially in the context of treating GI tumors. Oral administration prevents endotoxins from entering the circulatory system, at the same time, it can be in direct contact with the GI tumor. To sum up, although a series of trials evaluating the biosafety of OMV have been performed in animal models, there is still a lack of clinical research in humans. Moreover, determining the optimal safety and effectiveness tradeoff of OMVs remains one of the most challenging problems.

### 2.2 Engineered OMV-mediated delivery of anti-tumor drugs

OMVs were initially known to transport various biomolecules, such as nucleic acid, virulence factors, as well as proteins between cells. One of the core functions of OMVs better than other transport systems is their ability to carry several types of biomolecules simultaneously and prevent them from lytic enzymes over long distances transportation. This property of OMVs aroused the interest of OMVs involved in drug delivery systems in scholars. Initial studies focus on the use of OMV for drug delivery dating back to 1975. Since then, a steady stream of research has energized the field of OMV-mediated delivery of anti-tumor drugs 81.

#### 2.2.1 Delivery of chemotherapeutic agents by engineered OMVs

Chemotherapy has been the first therapeutic choice for most patients with GI tumors during the last decades. However, non-specific accumulation of chemotherapeutic agents in healthy tissues, instead of their targeted distribution, has diminished their application value and led to numerous side effects, such as cardiotoxicity, peripheral neurotoxicity, bone marrow suppression, hair loss, nausea, and vomiting 82. The intrinsic characteristics of certain chemotherapeutic drugs, like the high lipid solubility of paclitaxel and the facile degradation of camptothecin, also posed challenges to OMVs as drug carriers. Thus, researchers attempted to modify OMV to construct more stable and efficient drug delivery systems to improve the comprehensive effect of chemotherapy.

The *E. coli* strain *Nissle 1917 (EcN)*, one of the gut probiotics, prefers to proliferate at the interface between the necrotic and hypoxic regions of hosts, which enables them natural tumor-targeting potential. The derivates of *EcN*, OMVs, were also proved to deliver Anti-tumor drugs to the tumor hypoxic areas, thereby reducing the tumor burden of CRC mice 83. A comparative study showed that the chemotherapeutic drug Doxorubicin (DOX) loaded in OMVs exhibited better therapeutic response than that in liposomes or DOX alone in CRC. OMV-based drug delivery increased the half-life of drugs, decreased the clearance rate, and enhanced the bioavailability of the loaded drug 84. The additional anti-tumor response may be attributed to the immunogenicity-induced aggregation of macrophages and significantly elevated cytokine levels in tumor tissues.

Paclitaxel (PTX), primarily targets cellular microtubule proteins to inhibit the cell cycle and has been widely used in the treatment of GI tumors. When PTX is synergistically administered with *Salmonella typhimurium* OMVs, the anti-tumor effect is further enhanced in CRC and HCC patients. Besides inhibiting angiogenesis by downregulating VEGF expression, the OMV-enveloping drug increased tumor cell apoptosis and autophagy, and increased Natural killer cells (NK cells) infiltration in tumor tissues 85. The OMV-based platform enhances the efficacy of chemotherapy and minimizes its off-target and adverse effects. Solomon *et al.* utilized OMVs incorporated with PTX and attached bispecific antibodies (BsAb) to the OMV surface. When first used in clinical trials, the PTX-encapsulated OMVs were shown to be safe and well-tolerated in patients with advanced gastric, esophageal cancer, CRC, and PDAC. A significant secretion of the anti-tumor-associated cytokines (*e.g.*, TNF-α) was also observed in the serum of patients four hours after intravenous administration, which could partially explain the anti-tumor effect of the engineered OMVs 86. In addition to PTX, other traditional chemotherapeutic agents commonly used in the treatment of digestive system tumors, including hydrophilic (*e.g.*, irinotecan), hydrophobic (*e.g*., cisplatin, carboplatin), or amphiphilic (*e.g*., DOX, vinblastine, and 5-fluorouracil) drugs, have been successfully incorporated into engineered OMVs. Engineered OMVs have been reported to transport chemotherapeutic drugs in a manner that greatly reduces the concentration of required chemotherapeutic agents and the accumulation of chemotherapeutic agents in normal tissues 87.

**Figure 5:**
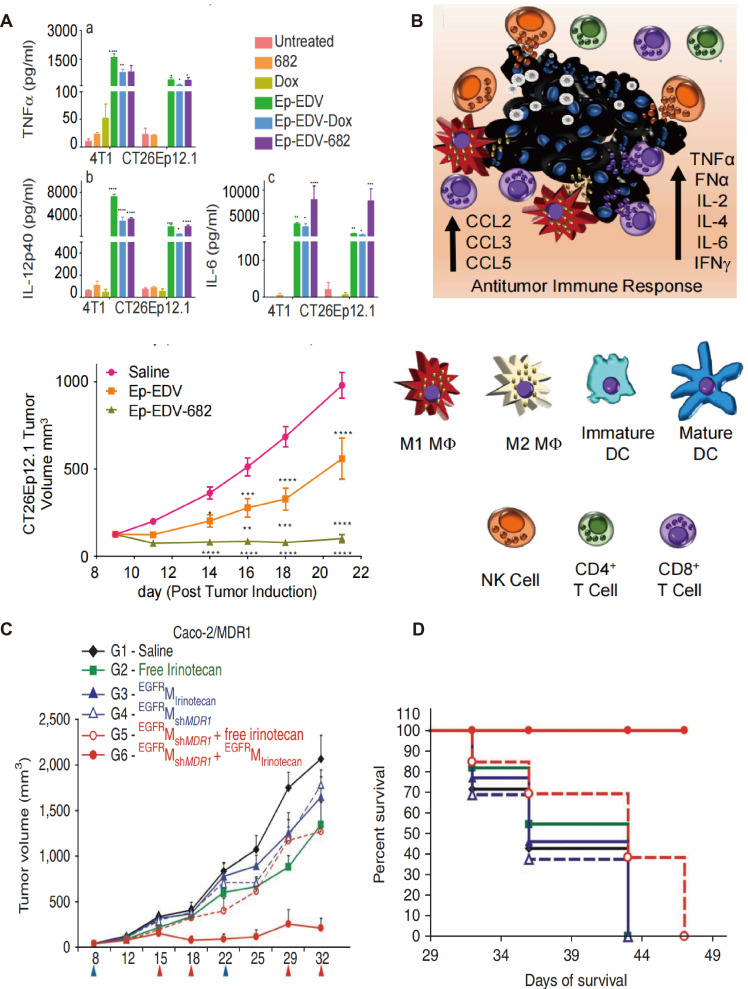
Anti-tumor effects of engineered OMVs with optimized immunogenicity. A-B) Schematic of the proposed mechanism of engineered OMVs reduced tumor volume with increasing concentrations of TNF-ɑ (a), IL-12p40 (b), and IL-6 (c), as well as growth inhibition in CT26 tumors. Adapted with permission from 96, copyright 2020 Cancer Cell. C) Drug resistant Caco-2/MDR1 xenografts were treated with sh*MDR1*-loaded OMVs followed by irinotecan-loaded OMVs on days shown below the x-axis, which resulted in significant antitumor effects. D) Kaplan-Meier survival analysis showing 100% survival only in the mice receiving the sequential treatment of sh*MDR1*-loaded, EGFR presenting OMVs and irinotecan-loaded ^EGFR^OMVs. Reproduced with permission from reference. Adapted with permission from 97, copyright 2009 Nature Biotechnology.

Engineered OMVs also offer a promising platform for the delivery of more potent and cytotoxic drugs that cannot be used as conventional chemotherapeutic agents. PNU-159682, a metabolite of Anthracyclines and a DNA topoisomerase I inhibitor, overcomes multiple resistance mechanisms in tumor cells. However, it is about 2000-fold more toxic than the conventional drug Adriamycin, which precludes its clinical application 88. Sagnella *et al.* developed a drug delivery system incorporating PNU-159682 into *S. Typhimurium* OMVs, which were administered to mice with CRC. Engineered OMVs were modified with EGFR ligands on their surface in this study to improve their tumor-targeting properties. The new system exhibited significant anti-tumor effects without any apparent adverse effects. A schematic of the proposed mechanism of engineered OMV-induced activation of the immune system has been presented in Figure 5A. Anti-tumor related cytokines have significantly increased in the TME in Ep-EDV-682-treated CRC mice groups, which are known to promote both DC maturation and NK cell activation thus triggering a cascade releasing of cytokine production, resulting in a stronger cytolytic function. Notably, this engineered OMVs was further administrated in patients with advanced PDAC. CA19-9, and CRP levels were significantly decreased in the peripheral blood after the 12^th^ dose, and no significant adverse reactions were observed during treatment 89. This is one of the few engineered bacterial OMVs that have been used in GI tumor patients with therapeutic effects.

Engineered OMVs as carriers of chemotherapeutic drugs have higher drug loading capacity compared with traditional nanocarriers, which largely improved the efficiency of drug delivery. As reported, one OMV can encapsulate 10 million drug molecules at most. PTX shows a favorable binding affinity with OMVs compared to liposomes, because of the hydrogen bonds between PTX and DD-transpeptidase, the teichoic acid of the bacterial OMVs. Similarly, the quantitation of DOX packaging in OMVs showed approximately 100-fold higher than liposomes under the same co-incubation conditions 90. Except for internal drug loading, a small number of drugs can be genetically engineered onto the surface of OMVs. As the study reported by Ren *et al*., Polybia-mastoparan I, which exhibits preferential toxicity to tumor cells while having little damage to nontumorigenic cells, has been engineered onto OMVs surface with enhanced anti-tumor immune responses 91.

#### 2.2.2 Delivery of siRNAs by engineered OMVs

Short interfering RNA (siRNA), short hairpin RNA (shRNA), or micro RNA (miRNA) are subsets of double-stranded RNAs that effectively silence the expression of target genes at specific sequences, a process referred to as RNA interference (RNAi) in molecular biology 92. In cancer therapy, these RNAs have emerged as a powerful tool to inhibit GI tumor progression by downregulating pro-oncogenes, such as *Nuf2*, *Rap80*, *Hif-1α*, and *Vegfa* 93. However, the clinical use of siRNAs is limited due to their poor membrane permeability and stability, making them hardly cross the biofilm barrier and easily degraded by serum nucleases. Jivrajani *et al.* packed shRNAs targeting *Vegfa* in OMVs surface-modified with folic acid to treat GI tumor Xenograft mice. The engineered OMVs silenced the mRNA and protein expression of *Vegfa* in tumor tissues, and inhibited neovascularization to block the supply of oxygen and nutrients to tumors, eventually leading to tumor stabilization and regression 94. The combination of engineered OMVs containing therapeutic siRNAs and cytotoxic drugs may improve treatment efficacy and enhance susceptibility to chemotherapeutic agents. Jenner *et al*. demonstrated that the engineered OMVs delivering specific siRNAs against multidrug resistance genes (*e.g.*, *Plk1*) led to the elimination of previously chemoresistant tumors in an animal model of CRC 95. The positive results showed the potential of OMV as a platform for siRNA-based combination therapy. Combination therapy involving lower concentrations of chemotherapeutic drugs, siRNAs, and antibodies delivered by engineered OMVs offers a promising strategy to overcome the limitations of conventional systemic therapy. As Figure 5B presented, OMVs surface was modified in another study with EGFR-specific ligands and internally loaded with siRNAs against cell cycle-associated proteins, which have produced significant tumor growth inhibition in colon cancer models. Further, engineered OMV-based dual sequential treatment of shRNA and irinotecan reversed the chemoresistance and acquired improved survival benefit in GI tumor patients 36.

Currently, electroporation is a prevalent technique for introducing siRNAs into OMVs. Electroporation applies short, high-voltage pulses to induce the formation of transient pores in the cell membrane, creating a temporary state of permeability that enables the uptake of drugs, fluorochrome compounds, and large molecules such as nucleotides. Following the completion of the electroporation process, the phospholipid membrane undergoes structural reorganization without any permanent damage 98. Electroporation was first used to modify bacterial OMVs in 2014 39, which inspired the following studies to use this technique to introduce nucleotide cargoes into OMVs. Guo *et al.* add the specific siRNAs into OMV with electroporation, which targets the Redd1 metabolic pathway in tumor-associated macrophages. This novel approach significantly increased the infiltration of M2-type macrophages in tumor tissues and the levels of anti-tumor-associated cytokines, ultimately improving the survival rate of tumor-bearing mice 99. It is worth noting that different bacteria and drug payloads require precise regulation of electroporation parameters to avoid destabilizing the OMVs membrane 100. Besides internal drug loading, drugs can also be inserted into the membrane depending on lipophilicity or hydrophobicity.

### 2.3 Engineered OMV-based biomimetic NPs for GI tumors

Nanomaterials have emerged as important candidates for biomedical applications owing to their unique characteristics, such as naturally optical, electrical, magnetic, and electrochemical properties 101. However, the high cost of certain NPs (*e.g.*, gold) limitations in mass production, complex process of design and synthesis, and unproven biocompatibility and safety have restricted the clinical translation of NP-based drug delivery systems. OMV can be a great ally for improving NPs selective cell penetration, it can also prevent the immune clearance of NPs in clinics due to its good natural properties 90. The optimized pharmacokinetic properties of OMV-NPs have popularized their application in biomedical fields. The ain techniques of engineering OMVs with classical NPs and their applications in GI tumor treatment are described below.

#### 2.3.1 Gold NPs

Gold NPs have been widely used throughout the medical field because of their excellent stability. Gold NPs could improve the magnetic resonance imaging (MRI) contrast sensitivity of esophageal and gastric cancers without any apparent side effects, thereby significantly enhancing the tumor detection rate 86. Gold NPs offer a stable physical environment that facilitates electron transfer between electrode surfaces in contact with them and preserves protein bioactivity when bound to proteins. Additionally, they directly couple and interact with a broad range of molecules such as proteins, drugs, antibodies, enzymes, nucleic acids (DNA or RNA), and fluorescent dyes. Thus, they are considered versatile platforms for drug loading and targeted delivery. Gold NPs have also been utilized as targeted labeling agents for GI tumor tissues 102. In addition to their application in diagnostics, gold NPs are a good platform for drug loading and targeted delivery. Given their tuneable and unique optical properties, gold NPs have attracted widespread interest as mediators of noninvasive radiofrequency ablation and photothermal therapy (PTT) for improving treatment efficacy in GI tumors. As previous literature reported, gold NP-gemcitabine complexes can reverse chemotherapy resistance in PDAC and significantly improve patient survival 88. Their tunable and unique optical properties have attracted widespread interest in their use as mediators of non-invasive radiofrequency ablation and PTT in GI tumor therapy 103.

Recent studies have explored the use of gold NPs combined with biological materials, such as cell membranes, bacterial extracellular vesicles, and antibodies, to further functionalize gold NPs for optimized efficacy. Piao *et al.* used erythrocyte membranes to encapsulate gold NPs and found them to prolong circulation time and tumor targeting in both PTT and chemotherapy 104. Recently, engineered OMVs have been shown to be more promising biomimetic materials for NP modification, as they are inexpensive, easily editable, and biocompatible. Gao *et al*. used *E. coli* OMVs encapsulated with gold NPs to form biomimetic NPs with a size of 30 nm and used them as tumor vaccine adjuvants. These biomimetic NPs triggered T-cell responses in *vivo by* inducing the production of INF-γ and IL-17. In addition, the biomimetic NPs enhanced the activation and maturation of DCs in lymph nodes and generated humoral immune responses with higher affinity 105. OMV was also utilized to establish a platform to combine NP with another therapeutic agent. In a preclinical study, the *E. coli* OMV-based biomimetic Gold NPs were cleverly constructed for synergetic PTT and immunotherapy. The modified NPs enhance macrophage chemotaxis by upregulating TNF-α expression and induce the accumulation of reactive oxygen species (ROS) after low-dose laser irradiation, resulting in strong anti-tumor effects and improved survival of tumor-bearing mice 106.

#### 2.3.2 Mesoporous silica NPs

Mesoporous silica nanoparticles (MSNPs) have garnered significant attention as potential drug delivery vehicles for the treatment of GI disorders, particularly inflammatory bowel disease (IBD), due to their desirable characteristics such as controlled particle size, high specific surface area, low toxicity, hemocompatibility, and ease of surface modification 107. MSNPs achieve site-specific and controlled release of guest drugs from pore channels with external triggering motifs. Whereas, MSNPs may be destabilized in saline buffers to form aggregation, thus leading to the physically adsorbed drugs' premature release. To improve the drug-loading stability as well as the tumor-targeting ability of MSNPs, researchers explored OMV to wrap MSNPs for immune evasion. Covering of OMV improved MSNP biocompatibility, which can be explained by phagocytosis and delivery of OMV by neutrophils 108. In a study on CRC, MSNPs that loaded with DOX internal were also surface-decorated with *E. coli* OMVs. OMVs as biological outer membranes help the drug delivery system adsorb on the intestinal mucosa to target tumor cells, which is responsible for not only improved therapeutic effect but also reduces the leakage of DOX. In another study, Shi *et al.* treated CRC cells with 5-Fluorouracil (5-FU) loaded MSNPs, which were encapsulated in engineered *E. coli* OMVs 109. This delivery system profits from the high drug-loading capacity of MSNPs as well as the intestinal absorption ability of OMV via a specific Hyaluronic Acid (HA) receptor. The modified carriers enabled specific targeting of the tumor site and provided a viable strategy to improve the oral bioavailability of 5-FU, leading to effective tumor growth inhibition while minimizing side effects 110.

#### 2.3.3 Ferrosoferric oxide -Manganese dioxide (Fe_3_O_4_-MnO_2_) NPs

It has been reported. that systemically or locally administered iron oxide nanoparticles inhibited cancer growth by inducing a pro-inflammatory immune response with M1 macrophage polarization besides ferroptosis 111. Nevertheless, iron oxide nanoparticles triggered ROS-induced tumor damage may be resisted by the intracellular redox balancing mechanisms, limiting the therapeutic efficacy of ferrotherapy alone. Assisting materials therefore need to be applied to overcome the role of this endogenous balance. MnO_2_ is a widespread multifunctional therapeutic agent for relieving tumor hypoxia and improving tumor treatment efficacy. The structure of MnO_2_ includes Nanospheres, nanosheets, as well as hollow nanoparticles. Liu* et al.* deposited MnO_2_ with Fe_3_O_4_ in the envelopes of OMVs from* E. coli.* Taking Fe_3_O_4_ as PTA, they utilize *E. coli* OMVs as peroxidase carriers to relieve tumor hypoxia, thus Mn2+ activates ICD effects through glutathione (GSH)-consumed ferroptosis as well as the generation of ROS. The immune-stimulated OMVs hitchhike on the circulating neutrophils, resulting in improved tumor targeting. Finally, the engineered OMV produces stronger anti-tumor immune responses and enhances the therapeutic effect 112.

### 2.4 Engineered OMV-mediated photothermal therapy for GI tumors

**Figure 6:**
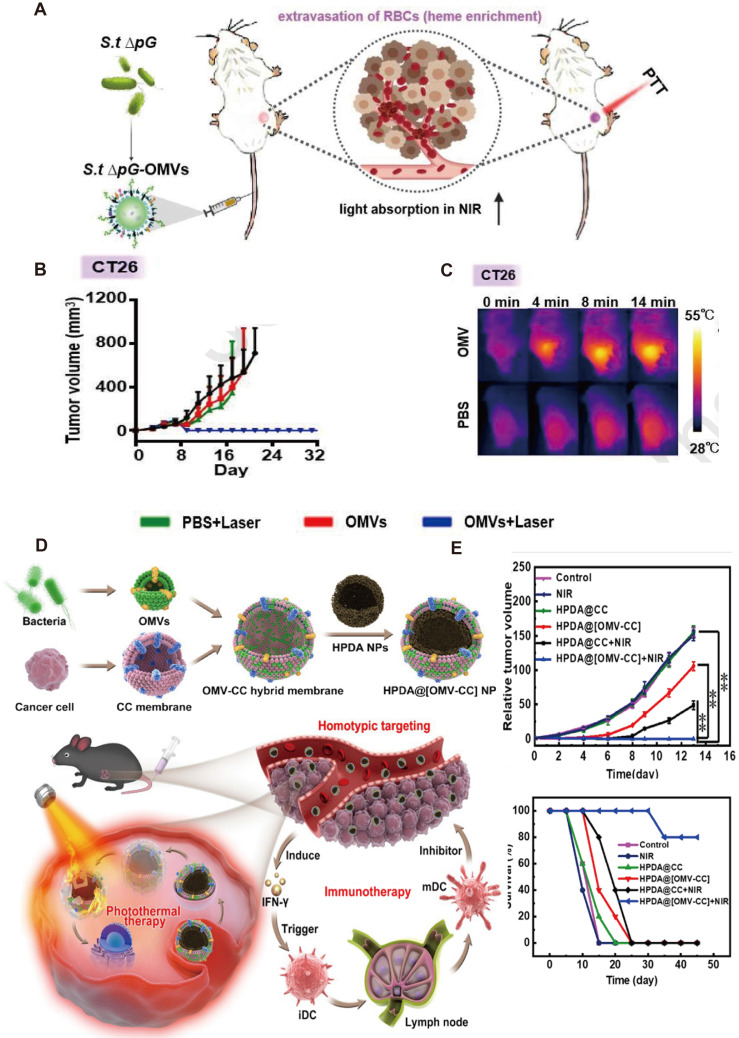
Anti-tumor effects of engineered OMV-based PTT. A) Intravenous injection of engineered OMVs could lead to extravasation of red blood cells in the tumor, further enabling effective photothermal ablation of the tumor by the NIR laser. B) IR thermal images and the corresponding time-dependent tumor temperature changes of CT26 tumor-bearing mice under NIR laser irradiation post injection of PBS or OMVs. C)Body weights of CT26 tumor-bearing mice post different treatments as indicated. Adapted with permission from 120, copyright 2021, Biomaterials. D) Schematic of the membrane derived from OMV and CC fusion and the resulting engineered OMV-CC camouflaged HPDA NPs to produce HPDA@[OMV-CC] NPs. E) Synergistic Photothermal/Immunotherapy of engineered OMVs and the relative tumor volume and long-term survival of mice after different treatments. Reproduced with permission from reference. Adapted with permission from 116, copyright 2020, Applied Materials.

PTT involves the use of photosensitizers (PSs) with high efficiency of photothermal conversion to convert light energy into heat energy to kill cancer cells. This approach induces the release of tumor antigens from heat-damaged cells, leading to the activation of tumor-specific CTLs, which mediate anti-tumor immune responses. Compared with traditional therapeutic modalities for GI tumors, PTT is relatively non-invasive and less toxic. However, the heterogeneity of tumor antigens, the complexity of laser dosimetry, and the potential damage of tumor surrounding tissue damage constrain its broad application 113. It is therefore of great importance to come up with combinatorial therapeutic methods to improve the PTT efficacy. A study on CRC showed that *Salmonella*-derived OMVs systemic administration induced tumor site inflammation, then causing erythrocyte leakage, which increased the absorbance of the near-infrared (NIR) laser in tumor tissues. After low-dose laser irradiation, neither tumor recurrence nor metastasis was observed besides significant tumor regression. Compared to conventional PTT, the OMVs-combined strategy reduces the required doses of PSs and NIR and enhances tumor-targeting and killing activities (Figure 6A). Zhuang* et al.* found that intravenous injection of *Salmonella typhimurium* OMVs significantly increased NIR optical absorption of GI tumors without adding any exogenous PSs 114. To take advantage of the membrane stability of OMVs, some biologically active molecules, such as enzymes, can be loaded into OMVs to inhibit tumor growth after laser irradiation. Engineered OMVs have demonstrated the potential to achieve precise targeting of tumors by exploiting the host immune response. Zhang* et al.* assembled hydrophobic PSs Ce6 with catalase (CAT) and loaded it into bacterial OMVs surface-modified with PD-L1. Relative to CAT-Ce6 alone, that modified with bacterial OMVs resulted in higher intratumor drug concentration and smaller tumor volume after laser irradiation 115.

The recent emergence of membrane fusion approaches facilitated PTT development. On the one hand, the incorporation of tumor cell membranes into OMVs prevents the loss of tumor antigens. On the other hand, the introduction of OMVs enhances the recognition of tumor membrane antigens by the immune system. Wang *et al.* fused bacterial OMVs with tumor cell membranes to obtain OMV-CC hybrid membranes and successfully coated them on hollow polydopamine NPs (Figure 6B). Combined with OMVs, polydopamine NP-mediated PTT activated immune responses in tissues of tumor and resulted in enhanced anti-tumor effects. On intravenous administration, these biomimetic NPs can homogeneously target tumor tissues and activate immune responses by rapidly stimulating DC maturation in lymph nodes. The anti-tumor immune response and PTT mutually enhance the therapeutic effects to completely eradicate tumors without causing obvious adverse effects 116. Synthetic OMV that integrates immunogenicity, cargo encapsulation, and photothermal conversion effect combined with other therapeutic methods has been raised to improve the therapeutic efficacy of PTT. For example, Zhai* et al*. constructed fusion bio-nanocarrier liposomes by fusing OMVs with photothermal-sensitive liposomes (PTSLs). High expression of CD38 on T cells in TME is associated with the immunosuppression of tumors. The use of PTSLs to deliver CD38-targeting siRNAs and PD-1 monoclonal antibodies produced significant anti-tumor effects in CRC and HCC. They also observed CD8+ T-cell infiltration at both primary and metastatic tumor sites and increased levels of anti-tumor-associated cytokines 117.

As mentioned above, the gold NPs and Fe_3_O_4_-MnO_2_ have shown promise as PSs in the treatment of various GI cancers, including esophageal, gastric, biliary tract, and pancreatic cancer. However, it has been uncovered that only 0.7% of these PSs successfully target the tumor site since the rapid clearance by the mononuclear phagocytic system in animal models. OMVs were utilized to coat PS, thereby in *situ* hitchhike circulating neutrophils for PSs' tumor targeting transportation. Through this work, neutrophil-mediated intratumor accumulation of PSs was improved by 300 to 600% which could be attributed to OMVs-caused inflammatory chemotaxis, and immune evasion by cell membrane camouflage 118. In another research on CRC mice, OMVs were encapsulated into nano-long micelles that loaded with PSs to harness the immunogenicity of OMVs, which attracted neutrophils for phagocytosis 119. Upon subsequent irradiation of tumor tissue with infrared light, an inflammatory microenvironment was induced, further enhancing the tumor-targeting and killing efficacy of PTT. Li* et al.* presented a promising OMV-based vaccine that facilitates immune-mediated tumor clearance after PTT in *situ*. The *E. coli-*derived OMVs were surface-modified with maleimide (Mal) groups for tumor neoantigens capture. At the same time, the 1-methyl-tryptophan (1-MT) was loaded into the OMV-Mal to reverse the tumor immunosuppression. The anti-tumor effect of PTT combined with 1-MT@OMV-Mal was confirmed both in primary and distant lesions in murine CRC models 119. To sum up, the application of engineered OMVs in PTT warrants further investigation in future research.

### 2.5 Engineered OMV-mediated tumor vaccines for GI tumor

**Figure 7:**
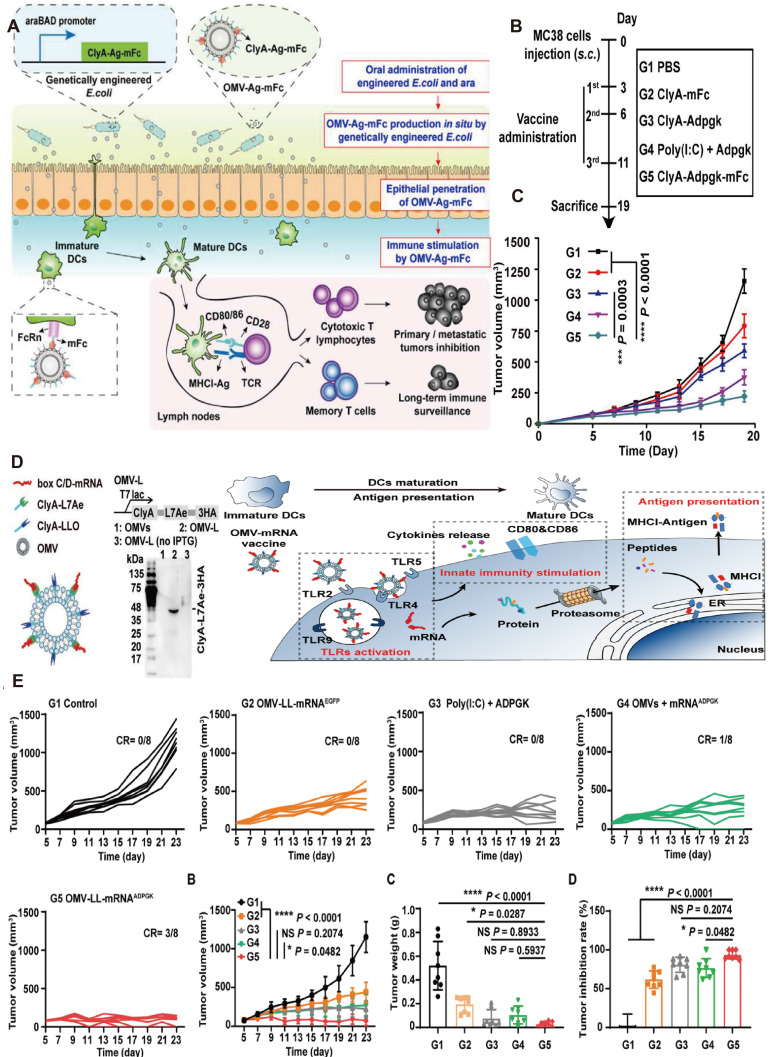
Engineered OMV-based oral tumor vaccine. A) Engineered *E. coli* were obtained by transformation with a plasmid expressing ClyA fused with a tumor antigen and Fc fragment of mouse IgG (ClyA-Ag-mFc). An arabinose-inducible promoter was introduced to control the fusion protein expression. OMV-Ag-mFc effectively penetrate the intestinal epithelial barriers, and are recognized and taken up by DCs in lamina propria. B) The schema showing the timeline of model construction and oral vaccination, and the tumor volumes were recorded every two days. Adapted with permission from 126, copyright 2022 Nature Biomedical Engineering. C) Schematic of the engineering OMV strategies. The RNA binding protein L7Ae and endosomal escape-promoting protein LLO were fused to the C-terminal of the ClyA surface protein on OMVs. D) The OMV-based mRNA vaccine triggering TLR activation, innate immunity stimulation, and antigen presentation. E) Growth curves of MC38 tumors bearing mice, growth curves of the average volumes, and the tumor weight of excised MC38 tumors have been recorded. Adapted with permission from 128, copyright 2022 Applied Materials.

Many tumor vaccines have been proven to be therapeutically efficient in animals, whereas only a few have been evaluated in clinical trials, which suggests that numerous problems remain to be overcome. One of the major limitations is identifying sufficiently immunostimulating tumor-specific antigens 121. Li *et al*. modified the *E. coli* ClyA sequence with the addition of the binding protein L7Ae and listeriolysin O (LLO), a protein known for promoting lysosomal escape. Followed by co-incubation, a therapeutic tumor vaccine that presents tumor-specific antigen mRNA was developed using OMVs as a vector (Figure 7B). Compared to free mRNA or liposome-based vaccines, the OMV-based mRNA vaccine notably facilitated DCs maturation, enhanced phagocytosis, and presentation of tumor antigens, and elicited stronger cellular immune responses, ultimately leading to the reduction of CRC tumor as shown as shown in Figure 7B. Remarkably, the vaccine demonstrated an anti-tumor immunological memory effect that persisted even 6 months after vaccination 122. Another limitation is finding a representative neoantigen that is co-expressed by all tumors is almost impossible, because of the heterogeneity of tumors 121. Zhao* et al.* evaluated the efficacy of two anti-tumor vaccines based on engineered OMVs. The first vaccine employed a 'tumor antigen plug-and-display procedure' in which protein tags were used to label tumor antigens, with the tags binding to OMVs expressing the 'catcher' for the efficient presentation of multiple tumor antigens on the surface of OMVs. The second vaccine was developed through a membrane fusion technique, where the fusion of tumor cell membranes and bacterial protoplasmic membranes onto poly lactic-co-glycolic acid (PLGA) NPs produced an immunogenic whole-tumor vaccine. Both vaccines elicited potent anti-tumor immune responses and demonstrated considerable tumor-killing activity in a mouse model of CRC. However, the former vaccine is insufficient in low mutation burden tumors, while the latter needs enough tumor tissue for vaccine synthesis, which can be obtained only through invasive procedures 123.

Additionally, tumor vaccines can be limited by the tumor burden-associated suppression of the systemic immune response 124. Surgical excision is contraindicated for advanced GI tumors due to its inability to improve survival and quality of life for patients. Research by Ma* et al.* proved that the debulking surgery for primary tumors before the vaccine could lead to a more satisfactory therapeutic effect in pancreatic cancer, hepatic cancer, and colon cancer mouse models. They explained that primary tumor resection has reversed the tumor-induced suppression of immune responses thus further preventing metastasis 124. Similarly, efferocytosis has been concluded as one of the mechanisms that contribute to tumor immunosuppression. Zhuang *et al*. engineered the OMVs for the efferocytosis blockade, tumor antigens capture, and presentation, consequently boosting the OMV-based vaccine efficiency 125.

The immune potency of the OMVs vaccine is influenced by the administration' s ways. Given that vaccines are injected intramuscularly or subcutaneously, OMVs-aroused anti-tumor response is generally restricted by limited lymph nodes and immune cells. Yue* et al.* recently devised an oral anti-tumor vaccine that can be controlled *in vitro* and relies on the capability of OMVs to traverse the intestinal mucosal barrier and interact with immune cells in the lamina propria, especially DCs. The oral administration of engineered bacteria and the expression inducer Arabinose (Ara) resulted in the production of OMVs expressing tumor antigens (OMV-Ag-mFc) within the intestine (Figure 7A). As Figure 7 showcased, OMVs-induced anti-tumor vaccine with oral administration has triggered anti-tumor immune responses and significantly suppressed tumor growth in murine models of CRC 54. Compared with subcutaneous or intramuscular tumor vaccines, oral administration has superior safety and patient compliance as well as reduced healthcare expenses.

Toxicity is generally considered one of the biggest hindrances to the clinical application of OMV-based vaccines. Several strategies have been addressed recently. First of all, developing new ways for vaccine administration, like oral administration. Without direct entry into blood circulation, oral delivery systems avoid cytokine storm induction, tissue extravasation, and reticuloendothelial system clearance, especially fit for the treatment of gastroenterological conditions, like GI tumors. Yue* et al.* used luciferase labeling to visualize the biodistribution and pharmacokinetics of OMVs. The results indicated that the bioluminescent signals are hardly detected in tissues other than those in the GI tract, which proved the biosafety of OMV-based oral vaccine (Accumulation in the cecum at 2 hours after administration, and gradually moved to the colon within 12 hours) 126. The epithelial cell penetration and immune activation of oral vaccines have also been well-proved. To prevent unexpected immune tolerance, Yue* et al.* also introduced an Ara-inducible promoter to guarantee the tumor antigen expression. Selection of certain strains, like probiotics or commensal bacteria seems to be another choice to avoid unwanted cytokine storm.

The *AKK* is a recognized intestinal probiotic. The protective effects produced by oral administration of *AKK* OMV on the gut barrier and commensal microbiota homeostasis were explored in the background of several GI tumor treatments. For instance, Shi* et al.* combined IL-2-based immunotherapy with* Akk* OMV, by which enhancing the anti-tumor immune response and tumor clearance 127. OMV derived from *Akk* was proven to enhance the immune activity of CTLs in CRC mice, whereas administration of *Akk* itself has no obvious influence on intestinal barrier integrity and gut homeostasis 10. In addition, ensuring dimensional uniformity with dynamic light scattering, as well as avoiding the interference of impurities during preparation are two other ways to reduce side effects. Apart from the above measures, specific elimination of LPS in OMVs by genetic engineering as well as membrane fusion with tumor cells are also feasible options 19. Overall, immunogenicity is the major source of OMV vaccine-induced adverse events and immunogenicity control is a double-edged sword because it may inhibit the anti-tumor effect. Investigations on methods to identify an ideal balance between low toxicity and high immunogenicity of OMV are still currently required.

## 3. Advantages of OMVs in GI Tumor Treatment

Different from other types of tumors, the GI mucosa possesses a large, orally accessible, and highly immunologically active interface, and thus represents attraction for tumor vaccine oral administration. Some studies have proved that mucosal immunizations are usually more effective than other parenteral routes, like subcutaneous or intramuscular injection in developing protective immunity to mucosal in GI tumors. GI tumors remain highly aggressive cancers, despite progress in therapy. Apart from conventional approaches, immunotherapy has become one of the primary treatment options for GI tumors especially those in advanced stages 129.

The intestine is considered the first immune organ of the human body, which is rich in both immune cells and mucosa-associated lymphoid tissues. High levels of T cells can be activated by mucosal immunostimulation in both the mucosal compartment and the related mesenteric lymph nodes 130. Then governing GI and the mucosal tumors through integrins-induced lymphocyte homing as well as proper cytotoxic activation within the tumor site, thus maximizing the efficiency of oral tumor vaccine. Karaki* et al.* found that only mucosal vaccination elicited CD8+ T cells expressing mucosal integrins (CD49a and CD103), which is crucial for intratumoral CD8+ T cell infiltration and tumor elimination 131.

The environment of the GI tract provided various natural triggers of OMV-based therapeutic systems like special pH, pressure, and temperature. Thus, therapeutic cargo can be released at target sites mediated by the specific bacteria or enzyme in the intended biological environments. Besides, OMV is superior to other drug carriers in protecting cargoes from harsh GI environment like gastric acids or lipases secreted by the pancreas and bile salts 132. Some probiotic OMVs were confirmed to penetrate intestinal physiochemical barriers, interact with specific epithelial cells, and even manipulate the gut microbiome proving alleviated primary resistance to PD-1 blockade in tumor immunotherapy 10.

## 4. Summary and future perspectives

Engineered OMV represents a promising nanoscale drug delivery platform due to its superior biosafety and capability to improve drug uptake and delivery efficiency. They can stabilize the transport of chemotherapeutic drugs, mitigate damage to normal tissues, and reduce drug leakage. Moreover, OMVs protect bioactive molecules (*e.g.*, enzymes, small-molecular proteins, and siRNAs) from degradation, which enhances the stability and functional completeness of the cargo. As membrane structures, OMVs can be readily processed and modified to enhance their existing properties or confer additional functions, such as targeting tumor cells, reversing TME immunosuppression, and activating an effective anti-tumor immune response. OMVs combined with organic or inorganic synthetic nanomaterials to achieve complementary advantages, which promotes the functionalization of OMV that improves the efficiency of therapy, and reduces the burden of purely synthetic nanomaterials mass production. It has been confirmed that the disturbed gut microbiota as well as their metabolites strongly affects the occurrence and the progression of GI tumor. Thus, the combination of OMV-regulated gut microecology with other types of therapy is a powerful strategy. In addition, oral administrated OMV contact with GI tumor is direct, without LPS entry into the bloodstream, and avoids the OMVs clearance by the reticuloendothelial system. This review has summarized the recent advances related to the application of engineered OMVs in the treatment of GI tumors. Approaches for engineering OMVs include genetic modification, internal drug loading, surface modification, and combination with NPs, *etc.* (Table 4). Owing to their unique advantages, engineered OMVs have garnered substantial attention from researchers, and numerous encouraging preclinical studies have been reported on their use in the past few years 133.

The intrinsic immunostimulatory property is the basis of engineered OMV in anti-tumor combinational therapies (Table 5). Nevertheless, OMV membrane substances cause excessive immune activation is still one of the most pressing concerns before clinical application. Developing multiple routes of administration or selecting OMVs derived from human commensal bacteria may become a new breakthrough. Cancer vaccines are primarily designed to stimulate tumor-specific immune responses through the delivery of tumor-associated antigens. OMVs, serving as immune adjuvants and tumor antigen carriers, represent promising candidates for tumor vaccine delivery. It is worth mentioning that suppressed tumor recurrence and metastasis have been observed in several OMV vaccine-mediated tumor treatments. It can be attributed to the long-term immune memory effects caused by the interaction of OMV-presented tumor-associated antigens tumor associated antigen with immune cells.

Although there are many advancements in engineered OMVs in the treatment of GI tumors, several obstacles must be overcome before their clinical translation. Apart from the potential immune-related adverse effects, significant problems including the relatively intricate and time-consuming isolation and purification procedures, as well as the unclear composition and mechanisms of OMVs are yet unsolved. Another challenge in the large-scale production of OMVs is the variability of batches due to the overexpression or knockout of specific genes, which affects the size, yield, and immunogenicity of OMVs. Thus, it is crucial to establish a standardized approach for the preparation of OMVs. To sum up, OMVs provide an excellent platform for integrating various therapeutic strategies for GI tumors. By engineering OMVs, personalized treatment strategies for GI tumors can be developed. Since engineered OMVs provide a platform to combine different therapeutic methods, optimizing the combination approach to achieve the most synergistic anti-tumor effect is a promising avenue for future research efforts.

## Figures and Tables

**Figure 8:**
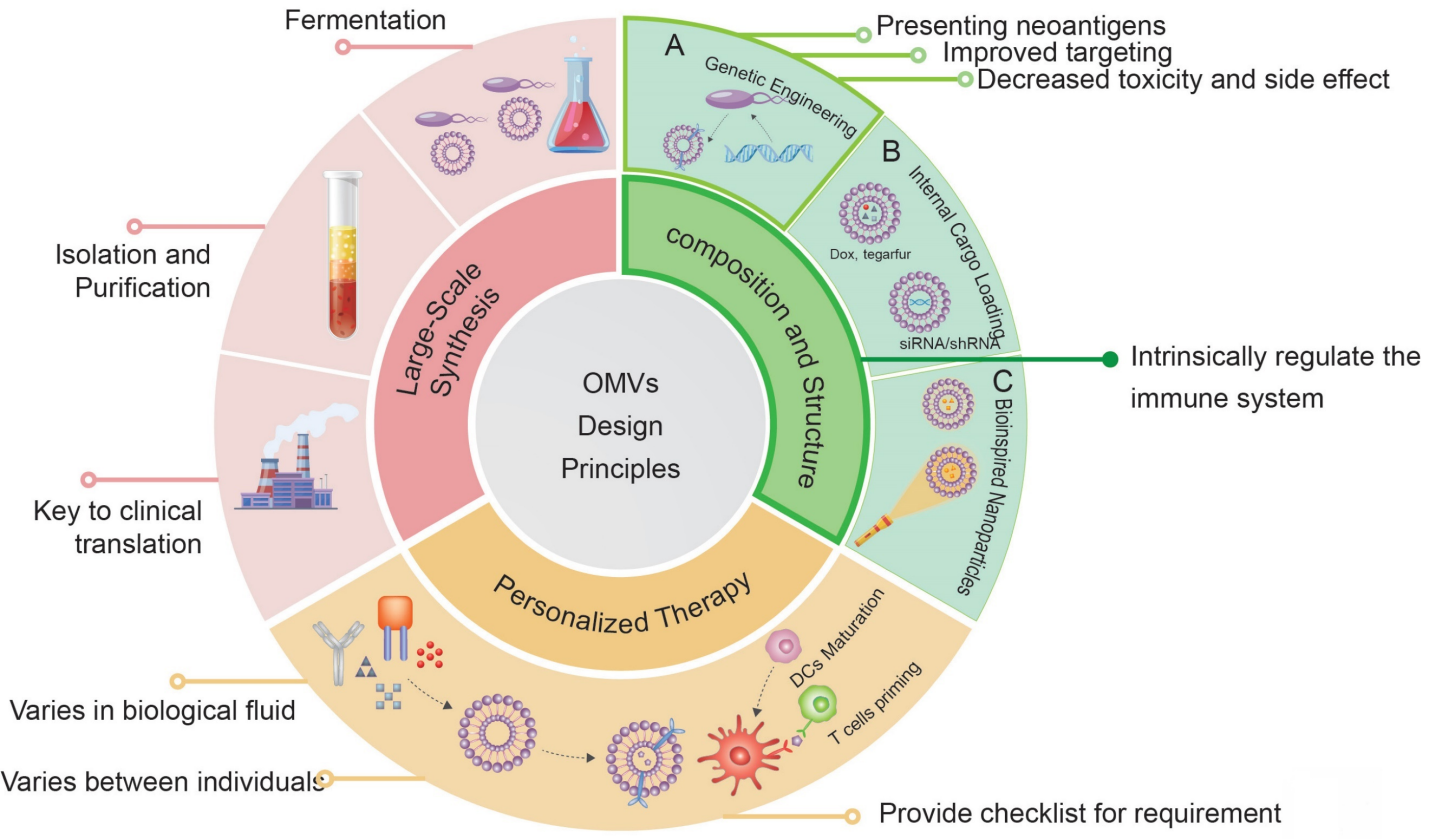
Design principles for engineered OMVs.

**Figure 9:**
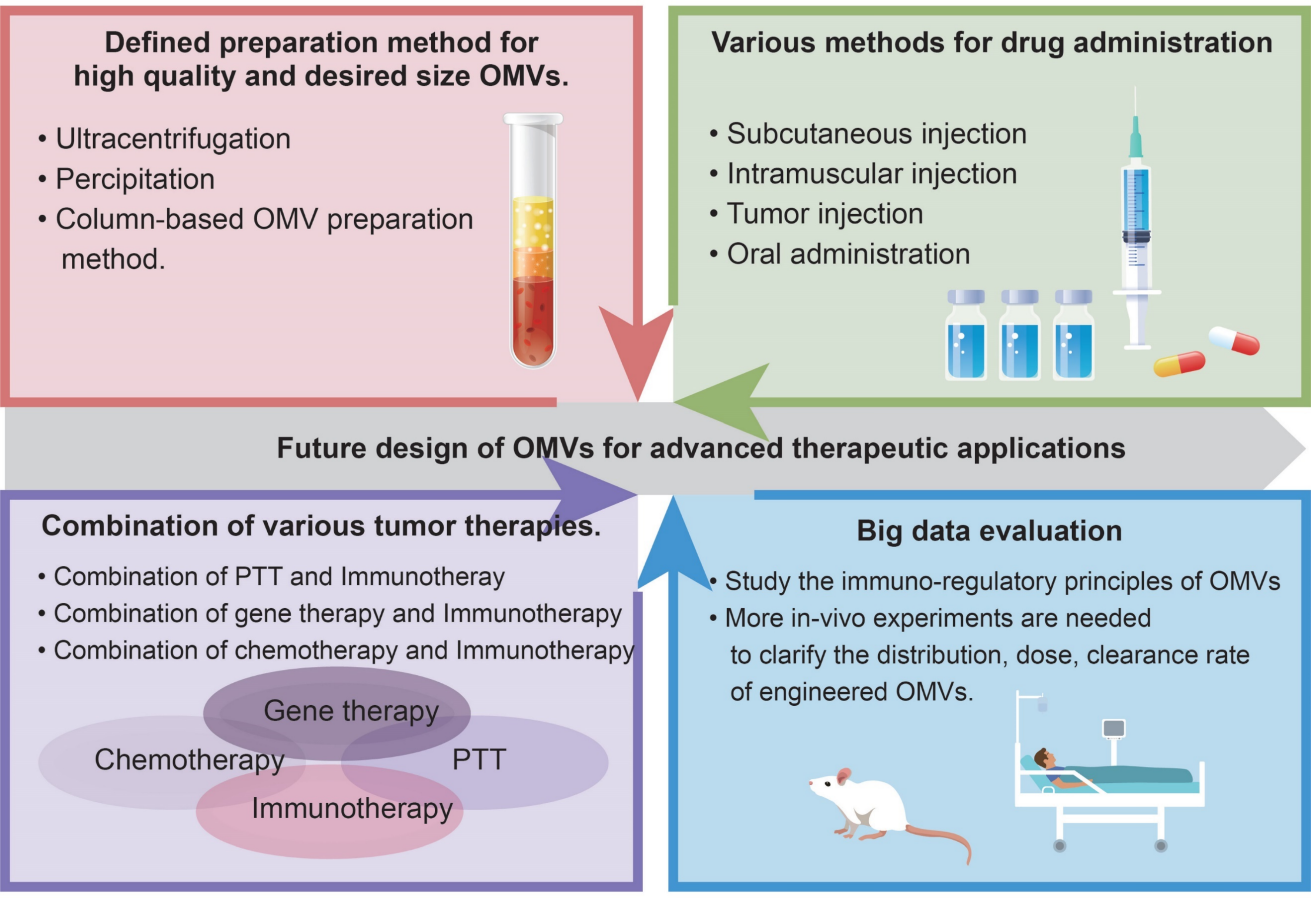
Future design of OMVs for advanced therapeutic applications.

**Table 1:** OMVs-induced immunomodulatory effects

Parental Bacteria	Immunomodulatory effects	Target cells	Reference
*Helicobacter pylori*	Dose-dependent IL-8 release	Gastric epithelial cells	30
*Escherichia coli*	Increased TLR-4 and IL-8 production	A498 and T-24 cells	31
*Helicobacter pylori*	Increased pro-inflammatory signal (NOD-1)	HEK 293 cells	32
*Neisseria meningitidis*	Increased IL-1b, IL-6, IL-8, IL-10, IL-12p40, TNF-α	Macrophages and monocytes	33
*Salmonella*	Increased TNF and NO	Mouse macrophages	29
*Salmonella*	Increased expression of CD86 and MHC-II. Increased release of TNF and IL-12	Dendritic cells	29
*Streptococcus*	The rapid uptake of MVs into DC2.4 cell lines. Increased release of TNF-α	Dendritic cells	28

**Table 2:** Genetic Modifications to Regulate OMVs Production

Parental Bacteria	Genetic modification	Description	Relative vesicular production	Reference
*Escherichia coli*	Δ*degP*, Δ*degS*, Δ*rseA*	Cpx, or σE pathways-deregulated membrane stress responses	Improved yield	52,53
	Δ*nlpI*	Deregulated anchored lipoprotein	Improved yield	53
	Δ*ompC*, Δ*ompR*, Δ*ompF*	Deregulated outer membrane porin	Improved yield	53
	Δ*pnp*	Deregulated polynucleotide phosphorylase	Improved yield	53
	Δ*ponB*	Peptidoglycan synthesis	Improved yield	53
	Δ*rmpM*	Outer membrane integrity	Improved yield	37
	Δ*tatC*	Inner membrane secretion apparatus	Improved yield	53
	Δ*tolA*, Δ*tolQ*, Δ*tolR*, Δ*tolB*	Outer membrane integrity and periplasmic protein	Improved yield	48,53
	Δ*waaG/rfaG*	ClyA biosynthesis; Glucosyl transferase	Improved yield	53
	Δ*wzxE*, Δ* wecF*	Inner membrane integrity	Improved yield	53
	Δ*yieM*	Unclarified	Improved yield	53
	Δ*ypjM*	Unclarified	Improved yield	53
	Δ*glnA*	Glutamine synthetase	Decreased yield	53
	Δ*lysS/herC*	Lysyl tRNA synthetase	Decreased yield	53
	Δ*nlpA*	Outer membrane lipoprotein	Decreased yield	53
	Δ*pepP*	Proline aminopeptidase	Decreased yield	53
*Acinetobacter baumannii*	Δ*BfmS*	Unclarified	Improved yield	50
	Δ*AbOmpA*	Outer membrane lipoprotein	Improved yield	54
*Campylobacter jejuni*	Δ*mlaA*	Lipid accumulation in the outer membrane	Improved yield	55
*Pseudomonas aeruginosa*	Δ*oprI*, Δ*oprF*	Outer membrane lipoprotein	Improved yield	56
*Serratia marcescens*	Δ*wecG*, Δ*wecD*	Decreased accumulation of lipid I at the inner membrane	Improved yield	57
*Bacillus subtilis*	Δ*xhlAB*/Δ*xlyA*	Unclarified	No effect	58
	Δ*ytCDEF*	Unclarified	No effect	58
*Streptococcus mutants*	Δ*sfp*	Phosphopantetheinyl transferase	Improved yield	59
*Mycobacterium tuberculosis*	Δ*pstA1*	Increased outer membrane lipoprotein	Improved yield	60,61
	ΔvirR	Unclarified	Improved yield	60
*Staphylococcus aureus*	Insert *Psmα*	Membrane integrity	Decreased yield	62
	Δ*tagO*, Δ*pbp4*	Decreased PG cross-linking	Improved yield	62
*Listeria monocytogenes*	Δ*sigB*	Membrane integrity	Improved yield	63
*Shigella sonnei*	Δ*tolR*	Inner membrane integrity	Improved yield	64
*Vibrio cholera*	Δ*vacJ*, Δ*yrbE*	Lipid accumulation in the outer membrane	Improved yield	65

Δ means gene deletion

**Table 3:** Genetic modification to regulate OMVs toxicity

Parental Bacteria	Genetic modulation	Effect on Lipid A	Outcome	Reference
***Escherichia coli***	Δ*lpx*, Δl*pxE*, Δ*LpxF*	Monophosphorylated lipid A	Decreased toxicity	72
***Salmonella typhimurium***	Δ*msbB*	Pentaacylated lipid A	Decreased toxicity	73
***E. coli K-12 W3110***, ***Salmonella typhimurium***	Insert *LpxR*	Pentaacylated lipid A	Decreased toxicity	74
***Neisseria meningitidis***	Insert *pagL*	Pentaacylated lipid A	Decreased toxicity	75
***E. coli K-12 W3110***	Δ*msbB*, Δ*pagP*	Pentaacylated lipid A	Decreased toxicity	76
***Escherichia coli***	Insert *lpxO*	Pentaacylated lipid A	Decreased toxicity	77
***Helicobacter pylori***	Insert *Hp0021*	Monophosphorylated lipid A	Decreased toxicity	78
***Helicobacter pylori***	Δ*LpxE*, Δ*LpxF*	Monophosphorylated lipid A	Decreased toxicity	79

Δ means gene deletion

**Table 4:** Engineered OMV-based tumor therapy

Type of engineering	Engineering strategy	Effect of modification	Effect of Engineered OMV-based tumor therapy	Reference
**Genetic** **Engineering**	∆*msbB*	Higher production yield OMVs with impaired lipidA	NK cells and T cells accumulate and produce IFN-γ and CXCL10 in the tumor	134
	∆*msbB and* fused ClyA with HA tags	Impaired lipidA, expression of the ClyA-mPD1E-3HA protein on OMVs	OMV-PD1 blocks the PD1/PD-L1 inhibitory axis, high levels of IFN-γ, IL-6, and TNF-α, NK cells, and CTL cell accumulation	40
	∆*msbB* and fused ClyA with HER2 affibody	OMVs expressed ClyA-Affibody recombinant protein	AffiHER2 OMVs were uptaken selectively by the tumor tissue	135
	*∆relA* and *∆spot*	OMVs with lower toxicity	Inflammatory and extravasation of RBCs in the TME, greatly enhanced tumor NIR absorbance	120
	Fused a plasmid expressing ClyA with a tumor antigen and Fc fragment of mouse IgG (ClyA-Ag-mFc)	Controllable production of OMVs loaded with tumor antigen (OMV-Ag-mFc) in the intestine	Inhibited the tumor metastatic and offering long-term protection	54
	Insert the gene fragments SpC or SnC into the plasmid	Engineered OMVs can display multiple tumor antigens	CD3+CD8+ T cells, CD3+CD4+ T cells, CD11b+Ly6G+ activated neutrophils, and CD11c+ DCs are all significantly elevated in the MC38 tumor microenvironment.	136
**Cargo Loading**	OMVs packed PNU-159682 by coincubation	OMVs loaded with 682 stably and modified with BsAb	M1 macrophage polarization, NK cell activation, CD8+ T cells generation	96
	OMVs capsulated MSN and 5-FU by high-pressure coextrusion	OMVs-MSN-5-FU	5-FU was delivered with tumor-targeting and reduced liver and spleen damage	110
	Tegafur was loaded into OMVs by high-pressure coextrusion	With immune modulation, lower drug leakage decreased side effects	A robust anti-tumor immune Memory generated	42
**Hybrid membrane**	OMVs and cancer cell membrane fusion by sonicating and extruding	Engineered OMVs present melanoma-specific antigens with high stability and low cytotoxicity	Engineered OMVs vaccination increases the CTLs level and CD4+ population, and suppresses tumorigenesis	137
	Fused OMVs with the cancer cell membrane by sonicating	Engineered OMVs present various tumor-specific antigens	The mice inoculated with CT26 tumor cells show complete tumor elimination and a tumor inhibition rate of 100%	136
	The PTSLs incorporated with Cypate fused with OMVs by coextrude	Cascaded double-target OMVs were constructed with the ability to carry the loaded cargos to the tumor tissues and target the T cells, respectively	Increased infiltration and cytotoxicity of T cells, improve the therapeutic effects of the PD-1 antibody	138
**Surface modification**	The DSPE-PEG-CA-PTX was escorted into OMVs by coincubation	Sequential pH-triggered prerelease of paclitaxel	Have satisfactory inhibition of tumor progression and metastasis	99
	OMVs fused with DSPE-PEG-RGD through the coextrusion	Improved blood circulation and tumor-targeting ability, decreased immunogenicity	A robust anti-tumor immune Memory generated	42

Δ means gene deletion

**Table 5:** Engineered OMVs involved in GI cancer

Parental Bacteria	Engineered strategy	Cancer type	Therapeutic strategy	Reference
***E. Coli W3110***	Gene engineering (PD-L1)	Colorectal Neoplasms	Immunotherapy	40
***E. Coli W3110***	Gene engineering (Decreased toxicity)	Colorectal Neoplasms	Immunotherapy	134
***E. coli Nissle1917***	Surface modification with ClyA (ClyA)-hyaluronidase (Hy)	Pancreatic and Colorectal Neoplasms	Immunotherapy	139
***E. Coli DH5α***	Hybrid membrane with cancer cells	Colorectal Neoplasms	Immunotherapy	140
***Salmonella Typhimurium***	Drug loading	Colorectal and Liver Neoplasms	Immunotherapy and chemotherapy	141
***Lactobacillus*** ***rhamnosus GG***	Gene engineering (Decreased toxicity)	Liver Neoplasms	Immunotherapy	142
***E. coli DH5α***	OMV-loaded micromotors	Colorectal Neoplasms	Immunotherapy	143
***E. coli BL21 (DE3)***	Drug loading	Colorectal Neoplasms	Immunotherapy and chemotherapy	144
***Salmonella Typhimurium***	Gene engineering (Decreased toxicity)	Colorectal Neoplasms	Immunotherapy and PTT	120
***S. Typhimurium*** ***minCDE- strain***	Drug loading	Colorectal Neoplasms	Immunotherapy and chemotherapy	89
***S. Typhimurium*** ***minCDE- strain***	Gene engineering (EGFR) and siRNA loading	Colorectal Neoplasms	Immunotherapy and Gene therapy	97
***Escherichia coli***	OMVs-MSNs-5-FU mesoporous silica	Colorectal Neoplasms	Immunotherapy and chemotherapy	110
***Salmonella***	Membrane fused with liposomes and siRNA loading	Colorectal Neoplasms	Immunotherapy and Gene therapy and PTT	138
***Escherichia coli***	Genetic engineering (fused the SpC with ClyA)	Colorectal Neoplasms	Immunotherapy and surgery	124
***E. coli BL21 (DE3)***	Gene engineering (CD47nb) and surface modification (PEG/Se)	Colorectal Neoplasm	Immunotherapy and radiotherapy	41
***Escherichia coli*** ***MG1655***	Drug loaded (UNC2025) and surface modification (Mal)	Colorectal Neoplasm	Immunotherapy and targeted therapy	114
***E. Coli***	Drug-loaded (IDO inhibitor) and surface modification (Mal)	Colorectal Neoplasm	Immunotherapy and PTT	145

## Abbreviations

OMVs: outer membrane vesicles
PDAC: pancreatic ductal adenocarcinoma
HCC: hepatocellular carcinomas
CRC: colorectal cancer
CAR-T: chimeric antigen receptor T-cell
GI: gastrointestinal
PD-1: programmed cell death 1
PD-L1: programmed cell death ligand 1
FDA: Food and Drug Administration
TME: tumor microenvironment
CTLs: cytotoxic T-cell lymphocytes
Akk: *Akkermansia muciniphila*
ICB: Immune checkpoint blockade
NPs: nanoparticles
LPS: lipopolysaccharides
OMPs: outer membrane proteins
Lpps: lipoproteins
PG: peptidoglycan
Tol-Pal: tolerance peptidoglycan-associated lipoprotein
PQS: pseudomonas quinolone sequence
ABC: ATP-binding cassette
*E. coli*: *Escherichia Coli*
*H. pylori*: *Helicobacter pylori*
*ETEC*: enterotoxigenic *Escherichia Coli*
PTE: phosphotriesterase
PRRs: pattern recognition receptors
TLR: Toll-like receptors
DCs: dendritic cells
APCs: antigen-presenting cells
MHC-I/II: major histocompatibility complex I/II
Cy7: Cyanine7
PTT: photothermal therapy
HER2: human epidermal growth factor receptor 2
ClyA: Cytolysin A
SIRPα: signal-regulatory protein alpha
SpT/SpC: SpyTag /SpyCatcher
fHbp: factor H binding protein
OVA: ovalbumin
VEGF: vascular endothelial growth factor
BFGF: basic fibroblast growth factor
PAMPs: pathogen-associated molecular patterns
EcN: *E. coli* strain *Nissle 1917*
NK cells: natural killer cells
BsAb: bispecific antibodies
siRNAs: short interfering RNAs
shRNAs: short hairpin RNAs
miRNAs: micro RNAs
RNAi: RNA interference
ROS: reactive oxygen species
MSNPs: mesoporous silica nanoparticles
IBD: inflammatory bowel disease
HA: hyaluronic acid
5-FU: 5-Fluorouracil
Fe_3_O_4_-MnO_2_: Ferrosoferric oxide-Manganese dioxide
GSH: glutathione
PSs: photosensitizers
NIR: near-infrared
CAT: catalase
PTSLs: photothermal-sensitive liposomes
Mal: maleimide
1-MT: 1-methyl-tryptophan
LLO: listeriolysin O
PLGA: poly lactic-co-glycolic acid
